# A simple constitutive framework for thinning–thickening transitions in dense granular–fluid mixtures

**DOI:** 10.1007/s11440-026-03242-9

**Published:** 2026-08-31

**Authors:** Yadong Wang, Wei Wu, Xiaogang Guo, Hongcheng Jiao

**Affiliations:** Institut für Geotechnik, Universität für Bodenkultur Wien (BOKU University), 1180 Vienna, Austria

**Keywords:** Dense granular flow, Granular suspension, μ(I) rheology, Shear thinning, Shear thickening

## Abstract

A unified constitutive framework is developed for dense fine-granular mixtures exhibiting apparent shear thinning and continuous or discontinuous shear thickening in the macro-viscous flow regime. The effective granular stress is decomposed into viscoplastic structural and suspension-stress contributions. The former is obtained from a hypoplastic model combined with a Herschel–Bulkley rheology, whereas the latter encompasses the $\mu (I_m)$ rheology and the Wyart–Cates frictional-contact transition, and shows stress-dependent jamming state. Benchmark calculations show that the proposed formulation captures the transition between shear-thinning and shear-thickening behaviour. A depth-averaged long-wave analysis further shows that non-monotonicity induced by discontinuous shear thickening may persist to promote instability in the macro-viscous flow regime.

## Introduction

Granular materials interacting with fluids are involved in a wide range of geotechnical and geo-environmental processes, including internal erosion, piping, sediment transport, debris flows, tailings transport, and fluid-driven particle migration in soils [20, 23, 26]. Depending on the solid fraction, confining stress, interstitial-fluid viscosity, and shear rate, a granular–fluid mixture may behave as a quasi-static granular skeleton, a viscous suspension, or a rapidly sheared granular flow dominated by particle inertia and collisions [4, 7, 8, 23, 58, 60, 64, 65]. After flow onset, such mixtures may further exhibit apparent shear thinning, continuous shear thickening (CST), or discontinuous shear thickening (DST) [9, 12, 37]. Developing a unified constitutive framework for these coupled responses remains challenging, especially for dense fine-granular mixtures in which structural resistance, viscous and collisional effects, and stress-activated shear thickening may coexist.

For relatively coarse granular–fluid mixtures, substantial progress has been made by describing the constitutive response through transitions among quasi-static, macro-viscous, and grain-inertial regimes [4, 33, 51, 53, 58, 60]. In such regime-based descriptions, a modified inertial number can be used to characterise the competition among the shear timescale, the particle-inertial timescale, and the viscous timescale of the interstitial fluid [2, 8]. The resulting friction and dilatancy laws compactly represent enduring frictional contacts, collisional momentum transfer, and macro-viscous resistance, forming a useful constitutive basis for coarse granular–fluid deformation [5, 55]. However, these formulations generally preserve a monotonic stress–shear-rate relation and do not explicitly account for the stress-activated transition from lubricated to frictional contacts once short-range interparticle repulsion is overcome [10]. This omission is particularly consequential for dense fine-granular suspensions, since DST-induced non-monotonicity can affect gravity-driven free-surface flows through inertia-free surface-wave instabilities and jammed-front-controlled propagation [7, 16].

Dense fine-granular suspensions may exhibit a sequence of regimes, including finite resistance near flow onset, apparent shear thinning, macro-viscous flow, and subsequent CST, DST, or shear jamming [12, 13, 18, 35]. These behaviours reflect the coexistence and competition of structural resistance and suspension-flow resistance. The structural resistance is associated with residual particle-skeleton strength and short-range attractive or structuring effects [24, 41, 49], whereas the suspension-flow resistance arises from viscous lubrication, hydrodynamic interactions, collisional effects, and stress-activated frictional contacts [34]. As the relative contribution of structural resistance decreases, the response becomes increasingly governed by the suspension-flow resistance [15]. At low applied stresses, particles may remain separated by thin lubrication layers or stabilising short-range repulsive forces, so that the suspension follows a relatively frictionless or weakly frictional branch. Once the applied stress exceeds a characteristic repulsive stress scale, direct frictional contacts are activated and may proliferate throughout the particle network [19, 31, 44]. The resulting frictional network increases the resistance to shear and drives the suspension towards a frictional branch with a lower effective jamming volume fraction [37]. A constitutive description must therefore accommodate both the low-rate structural response and the stress-activated suspension response.

Existing constitutive approaches describe different parts of this coupled problem [21, 46]. Elastoplastic models incorporating critical state provide a classical framework for describing rate-independent granular strength, dilatancy, and quasi-static shear deformation [29, 43, 45, 63, 66]. As an alternative route to critical-state soil modelling, hypoplastic models describe granular deformation through nonlinear stress-rate equations, without requiring an explicit yield surface, plastic potential, or flow rule [30, 40, 56, 57, 59]. Beyond quasi-static soil mechanics, related μ(I)-type and hybrid critical-state–μ(I)-type granular-flow formulations represent frictional and dilatant flow regimes through viscous, inertial, or mixed timescale ratios [3, 5, 23, 54, 55]. When supplemented by phenomenological yield-stress laws such as Bingham-type or Herschel–Bulkley-type descriptions [11, 15, 25, 39], these frameworks can represent residual strength and post-yield rate dependence, but not the stress-activated lubricated-to-frictional transition caused by overcoming short-range repulsion.

**Fig. 1:**
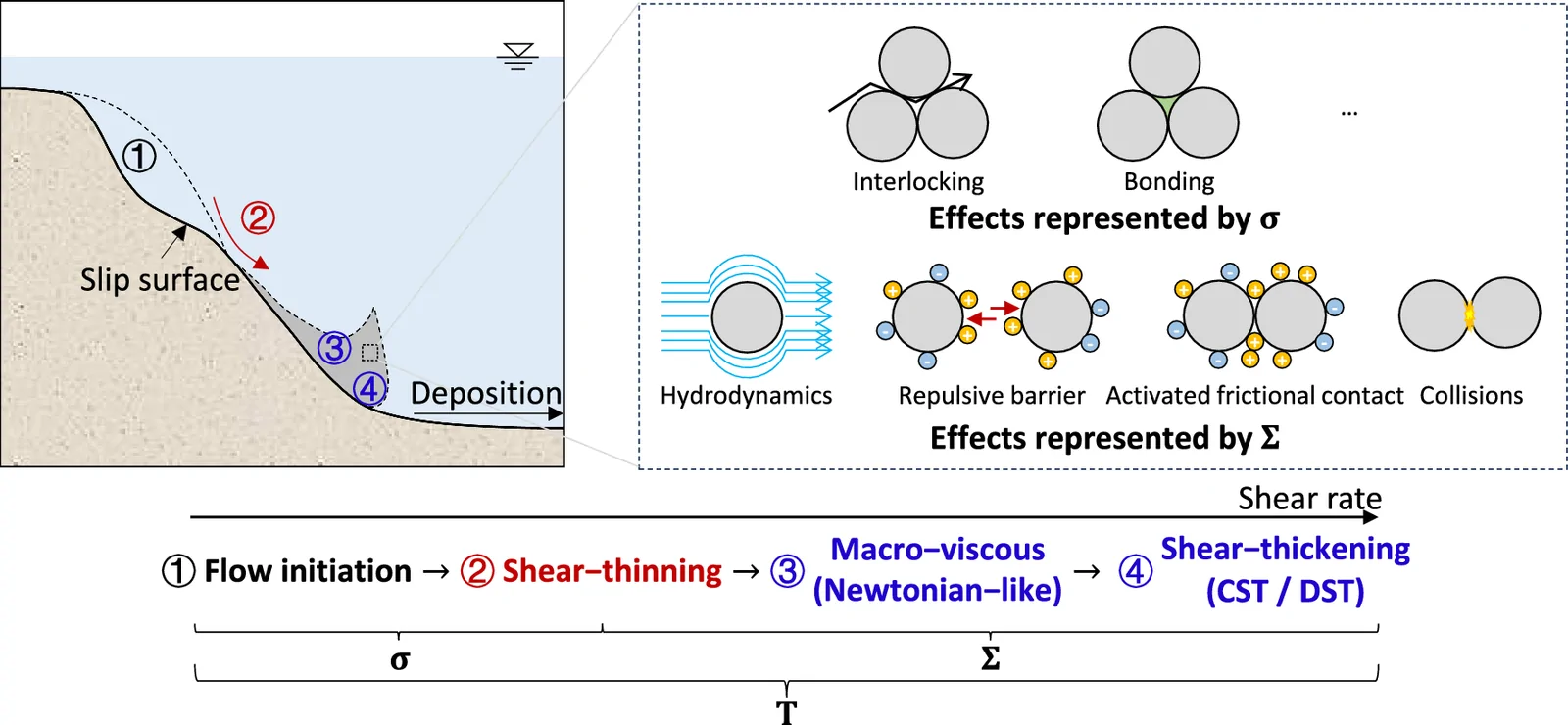
Conceptual illustration of the mechanical evolution of a fine-granular suspension from flow initiation to apparent shear thinning, macro-viscous flow, and subsequent shear thickening, together with the dominant interparticle interactions

By contrast, Wyart–Cates-type models and related suspension-rheology descriptions capture shear thickening through a stress-dependent fraction of frictional contacts and a corresponding stress-dependent jamming state [48, 61]. In many suspension-rheology descriptions, the low-stress branch is commonly treated as viscous, lubricated, or frictionless, whereas more general continuum mixture-theory formulations have incorporated frictional-contact evolution and granular skeleton effects in dense fine-particle suspensions [6]. For geomechanical granular–fluid mixtures, including debris-flow materials and soft Earth suspensions [28, 38], residual/yield structural resistance may nevertheless need to be retained, for example through a Herschel–Bulkley-type viscoplastic representation [14]. This is consistent with observations that finite yield stress in adhesive or cohesive non-Brownian suspensions can be governed by frictional constraints and shear-jammed microstructures [41, 49].

This study develops a simple constitutive framework for dense granular–fluid mixtures covering low-rate yielding, apparent shear thinning, macro-viscous flow, and shear-thickening transitions. The effective granular stress is decomposed into a viscoplastic structural stress, representing particle-skeleton resistance near flow onset, and a suspension stress incorporating viscous, collisional, and stress-activated frictional-contact effects. Apparent shear thinning is associated with the decreasing relative dominance of the viscoplastic structural stress, while CST and DST arise from the stress-activated evolution of the suspension stress towards a frictional-contact-dominated state through a stress-dependent jamming state. The formulation provides a unified constitutive description of thinning–thickening transitions in dense granular–fluid mixtures.

Section 2 introduces the mechanical picture and effective-stress decomposition, followed by the constitutive formulation and validation in Sects.  3–5. Section 6 examines a minimal depth-averaged free-surface application, and Sect. 7 summarises the main conclusions and limitations.

## Theoretical framework

### Mechanical picture of fine-granular suspensions

In the present work, a pragmatic size-based distinction is adopted for descriptive purposes [6]: mixtures with $d_s \gtrsim 100~\mu \textrm{m}$ are referred to as relatively coarse granular–fluid mixtures, whereas those with $d_s \lesssim 100~\mu \textrm{m}$ are referred to as fine-granular suspensions. This distinction is introduced solely to organise the benchmark materials considered here, and should not be interpreted as a universal regime boundary.

Fine-granular suspensions are idealised here as systems in which two effective resistance contributions coexist and compete: structural resistance and suspension-flow resistance, as presented schematically in Fig. 1. The structural resistance denotes the particle-skeleton resistance retained near flow onset, including residual or yield strength associated with unresolved contact, bonding, or structuring effects [14, 22, 42, 49, 62]. The suspension-flow resistance denotes the flow-induced resistance during shearing, including viscous lubrication, hydrodynamic interactions, collisions, and stress-activated frictional contacts after the repulsive barrier is overcome [44, 61].

Within this mechanical picture, an apparent shear-thinning branch is attributed to the decreasing relative dominance of the structural resistance as shearing develops, whereas CST and DST are attributed to the stress-activated growth of frictional-contact networks once the thin lubrication layers or stabilising repulsive barrier is overcome. This idealisation motivates the effective-stress decomposition introduced below, in which the structural resistance and the suspension-flow resistance are represented by separate stress contributions.

### Stress decomposition and effective granular stress

The total stress of the suspension is decomposed into a fluid contribution and an effective granular contribution [5, 53],

1 $$\begin{aligned} \begin{aligned} \textbf{T}_m = \textbf{T}_f + \textbf{T}\end{aligned} \end{aligned}$$

where $\textbf{T}_m$ is the total mixture stress tensor, $\textbf{T}_f$ denotes the viscous shear response of the fluid phase, and $\textbf{T}$ is the effective stress carried by the granular phase. This decomposition follows the classical Terzaghi’s effective-stress concept, while extending the fluid contribution beyond isotropic pore pressure to account for the viscous shear response of the fluid phase [5].

Related studies have shown that transitions between different rheological states may be described through alternative constitutive constructions [3, 6, 11, 39]. Here, following previous stress-decomposition formulations for granular–fluid mixtures [55, 58], the decomposition is chosen to mirror the two resistance mechanisms identified in Sect. 2.1: the structural resistance retained near flow onset and the suspension-flow resistance developed during shearing. The effective granular stress is therefore written as

2 $$\begin{aligned} \textbf{T}= \boldsymbol{\upsigma } + \boldsymbol{\Sigma } \end{aligned}$$

where $\boldsymbol{\upsigma }$ denotes the viscoplastic structural stress and $\boldsymbol{\Sigma }$ denotes the suspension stress. These terms are used as descriptive labels for two effective-stress contributions rather than as independently resolved microscopic force components. As defined in Sect. 2.1, the viscoplastic structural stress $\boldsymbol{\upsigma }$ represents the effective structural resistance retained by the particle skeleton near flow onset. This contribution is constructed from a critical-state reduction of the hypoplastic model and supplemented by a Herschel–Bulkley rheology. The suspension stress $\boldsymbol{\Sigma }$ represents the suspension-flow resistance and is formulated to incorporate viscous, collisional, and stress-activated frictional-contact contributions, with the latter governed by a stress-dependent frictional-contact state variable.

Building on this decomposition, the effective granular stress is proposed as

3 $$\begin{aligned} \textbf{T}= \underbrace{\eta _0 \dot{\textbf{e}} - \upsigma _{vol}\textbf{I}}_{\boldsymbol{\upsigma }} + \underbrace{\eta _1 \dot{\textbf{e}} - \eta _2 \dot{\gamma }^2 \textbf{I}}_{\boldsymbol{\Sigma }} \end{aligned}$$

where $\dot{\textbf{e}}$ is the deviatoric strain-rate tensor, $\dot{\gamma }=\sqrt{2\dot{\textbf{e}}:\dot{\textbf{e}}}$ is the shear rate, $\eta _0$ is associated with the viscoplastic structural stress, and $\eta _1$ and $\eta _2$ are the shear and volumetric coefficients of the suspension stress, respectively. In this form, the first two terms represent the viscoplastic structural stress that may remain active near suspension onset, whereas the latter two terms describe the suspension stress. The DST transition is introduced within the suspension stress through a stress-activated fraction of frictional contacts, which governs the evolution from a lubricated state to a frictional-contact-dominated state.

For the aligned stress contributions adopted in the present simple-shear formulation, the equivalent mixture shear stress can be written as

4 $$\begin{aligned} \begin{aligned} \tau _m = \sqrt{\frac{1}{2}\textbf{T}_{m,dev} :\textbf{T}_{m,dev} } =\tau _f + \tau _{\Sigma } + \tau _{\upsigma } \end{aligned} \end{aligned}$$

where the equivalent shear stresses associated with the structural and suspension contributions are defined as $\tau _{\upsigma } = \sqrt{\frac{1}{2}\boldsymbol{\upsigma }_{dev}:\boldsymbol{\upsigma }_{dev} }$ and $\tau _{\Sigma } = \sqrt{\frac{1}{2}\boldsymbol{\Sigma }_{dev}:\boldsymbol{\Sigma }_{dev} }$, respectively. The subscript *dev* denotes the deviatoric part of a tensor; for example, for a tensor $\textbf{A}$, one has $\textbf{A}_{dev} = \textbf{A} - \textrm{tr} \textbf{A} /3 \textbf{I}$. In the above equation, the term $\tau _f=\eta _f\left( 1 + \frac{5}{2} \Phi \right) \dot{\gamma }$ represents the equivalent shear stress associated with the viscous shear response of the fluid phase [17]. Based on Eq. (4), the apparent viscosity of granular–fluid mixtures is defined as

5 $$\begin{aligned} \eta _m = \frac{\tau _m}{ \dot{\gamma }} \end{aligned}$$

### Scope of constitutive analysis

The constitutive framework is developed under steady homogeneous simple shear at fixed volume. This idealised loading isolates the local constitutive response from geometric change, evolving stress paths, and boundary-induced heterogeneity [18, 44, 53], allowing the respective roles of structural resistance, suspension-flow resistance, and stress-activated frictional transition to be identified before addressing more general boundary-value problems.

## Constitutive formulation of the effective granular stress

The constitutive formulation of the effective granular stress is developed in this section based on the decomposition introduced in Sect. 2. Section 3.1 first recalls the previous formulation for relatively coarse mixtures as a baseline, followed by the formulations of the viscoplastic structural stress and suspension stress in Sects. 3.2 and 3.3, respectively. The former represents low-rate yielding and apparent shear thinning, whereas the latter incorporates stress-activated frictional contacts to capture CST and DST. Together, these two stress contributions provide a unified constitutive description of thinning–thickening transitions.

### Baseline formulation for granular–fluid mixtures

This subsection briefly recalls the constitutive formulation previously developed for relatively coarse granular–fluid mixtures [55], which serves as the baseline for the present unified framework.

In this baseline formulation, $\boldsymbol{\upsigma }$ was calculated using a hypoplastic model with critical state [56, 59]

6 $$\begin{aligned} \mathring{\boldsymbol{\upsigma }} = \mathbb {L}(\boldsymbol{\upsigma }):\textbf{D}+ f_e \textbf{N}(\boldsymbol{\upsigma }) \Vert \textbf{D}\Vert \end{aligned}$$

with

7 $$\begin{aligned} \begin{aligned} \mathbb {L}(\boldsymbol{\upsigma })&= f_s\Big [ \textrm{tr}\boldsymbol{\upsigma }\,\mathbb {I} + f_v \boldsymbol{\upsigma } \otimes \textbf{I}+\frac{a^2}{\textrm{tr}\boldsymbol{\upsigma }}(\boldsymbol{\upsigma }\otimes \boldsymbol{\upsigma }) \Big ] \\ \textbf{N}(\boldsymbol{\upsigma })&= f_s a\left( \boldsymbol{\upsigma }+\boldsymbol{\upsigma }_{dev}\right) \end{aligned} \end{aligned}$$

where $f_s$, $f_v$, and *a* are functions of Young’s modulus *E*, Poisson’s ratio ν, and internal friction angle ϕ. Their explicit expressions are given in [55]. The density function is defined as $f_e=e/e_c$, where $e_c = 1/\Phi _c - 1$ is the critical-state void ratio and $\Phi _c$ is the solid fraction at the critical state. $\mathring{\boldsymbol{\upsigma }}$ is the Jaumann stress rate. The rate-independent stress tensor can be calculated by $\boldsymbol{\upsigma } = \int \left( \mathring{\boldsymbol{\upsigma }} - \boldsymbol{\upsigma } \textbf{W}+ \textbf{W}\boldsymbol{\upsigma }\right) \textrm{d}t$, where $\textbf{W}$ denotes the spin tensor. The hypoplastic model has been implemented in numerical codes to solve boundary-value problems [36, 50, 52].

The viscosity-based suspension-stress tensor is given as

8 $$\begin{aligned} \boldsymbol{\Sigma }=\eta _{1}\dot{\textbf{e}}-\eta _{2}\dot{\gamma }^2\textbf{I}\end{aligned}$$

where $\eta _{1}$ and $\eta _{2}$ are the shear and volumetric coefficients, respectively. Their expressions can be found in our previous study [55].

The complete formulation given by Eqs. (6)–(8) serves as a constitutive basis for relatively coarse granular–fluid mixtures, accounting for internal friction and collisional interactions among solid grains, as well as the macro-viscous contribution of the interstitial fluid [55]. However, it does not explicitly account for the repulsion-controlled activation of frictional contacts that is central to fine-granular suspensions. The present formulation extends this constitutive basis to account for repulsion-controlled frictional activation, thereby enabling a unified description of both coarse and fine-granular–fluid mixtures.

### Viscoplastic structural stress

#### Proposed formulation of $\boldsymbol{\upsigma }$

The viscoplastic structural stress is introduced to retain residual or yield strength near suspension onset, particularly in dense granular mixtures. It extends the critical-state reduction of the hypoplastic backbone by a Herschel–Bulkley term and provides the structural stress used in the subsequent suspension formulation.

At the critical state, one has [56]

9 $$\begin{aligned} \mathring{\boldsymbol{\upsigma }}=\textbf{0}, \qquad \textrm{tr}\textbf{D}=0, \qquad f_e=1 \end{aligned}$$

Defining the normalised strain-rate tensor as $\textbf{n}=\textbf{D}/\Vert \textbf{D}\Vert$, it follows from $\textrm{tr}\textbf{D}=0$ that $\textrm{tr}\textbf{n}=0$ and $\textbf{n}:\textbf{n}=1$.

Substituting $\textbf{n}=\textbf{D}/\Vert \textbf{D}\Vert$ and Eqs. (9) into the hypoplastic Eq. (6) gives $\mathbb {L}(\boldsymbol{\upsigma }):\textbf{n} + \textbf{N}(\boldsymbol{\upsigma }) = \textbf{0}$. If $\mathbb {L}(\boldsymbol{\upsigma })$ is invertible, this yields $\textbf{n}=-\,\mathbb {L}(\boldsymbol{\upsigma })^{-1}:\textbf{N}(\boldsymbol{\upsigma })$. This relation gives the deviatoric stress tensor at the critical state as

10 $$\begin{aligned} \boldsymbol{\upsigma }_{dev} = \frac{3\upsigma _{vol}}{a}\,\textbf{n} = \frac{3\sqrt{2}\upsigma _{vol}}{a \dot{\gamma }} \dot{\textbf{e}} \quad \text {with} \quad a = \frac{\sqrt{3}(3 - \sin \phi )}{\sqrt{8} \sin \phi } \end{aligned}$$

Equation (10) shows that the deviatoric stress tensor is collinear with $\textbf{n}$ at the critical state. Since $\textrm{tr}\textbf{D}=0$, one has $\textbf{D}_{dev}=\textbf{D}$, and therefore, $\textbf{n}$ may be equivalently defined from either $\textbf{D}$ or $\textbf{D}_{dev}$. $\dot{\textbf{e}} = \textbf{D}- \textrm{tr}(\textbf{D})\textbf{I}/3$ denotes the deviatoric strain-rate tensor. $\dot{\gamma }= \sqrt{2 \dot{\textbf{e}}: \dot{\textbf{e}} }$ is the shear rate. The detailed derivation of Eqs. (9)–(10) and further discussion of the hypoplastic model with critical state are given in Appendix A.

To close the above expression, a constitutive relation for the mean pressure carried by the viscoplastic structural stress is required. Previous studies on dense granular–fluid mixtures and debris-flow-type materials have shown that a finite stress level may be retained near the onset of flow [55, 58], and that the post-yield shear response can often be represented by a Herschel–Bulkley-type relation [13, 15, 28, 38]. Motivated by these observations, the mean pressure associated with the viscoplastic structural stress is written as

11 $$\begin{aligned} \upsigma _{vol} = K_1 f_{\Phi } + K_2 f_{\Phi }\dot{\gamma }^{0.5} = f_{\Phi } \left( K_1 + K_2\dot{\gamma }^{0.5} \right) \end{aligned}$$

where

12 $$\begin{aligned} f_{\Phi } = \left( \frac{\Phi }{\Phi _{m}-\Phi } \right) ^2 \end{aligned}$$

Here, $f_{\Phi }$ describes the increase in structural resistance as the solid fraction approaches the jamming solid fraction associated with the structural resistance, denoted by $\Phi _m$. The coefficient $K_1$ controls the static residual or yield pressure, whereas $K_2$ controls the contribution of Herschel–Bulkley rheology. When $K_2=0$, Eq. (11) reduces to the purely residual pressure form used to represent the critical-state structural resistance [56].

Combining Eqs. (10) and (11), the viscoplastic structural-stress tensor can be written as

13 $$\begin{aligned} \boldsymbol{\upsigma } = \eta _0 \dot{\textbf{e}} - \upsigma _{vol} \textbf{I}\end{aligned}$$

where $\eta _0 = 3\sqrt{2}\upsigma _{vol} /(a \dot{\gamma })$. Here, $\boldsymbol{\upsigma }$ should be interpreted as a viscoplastic structural stress including both a static residual component and a Herschel–Bulkley rheology.

**Fig. 2:**
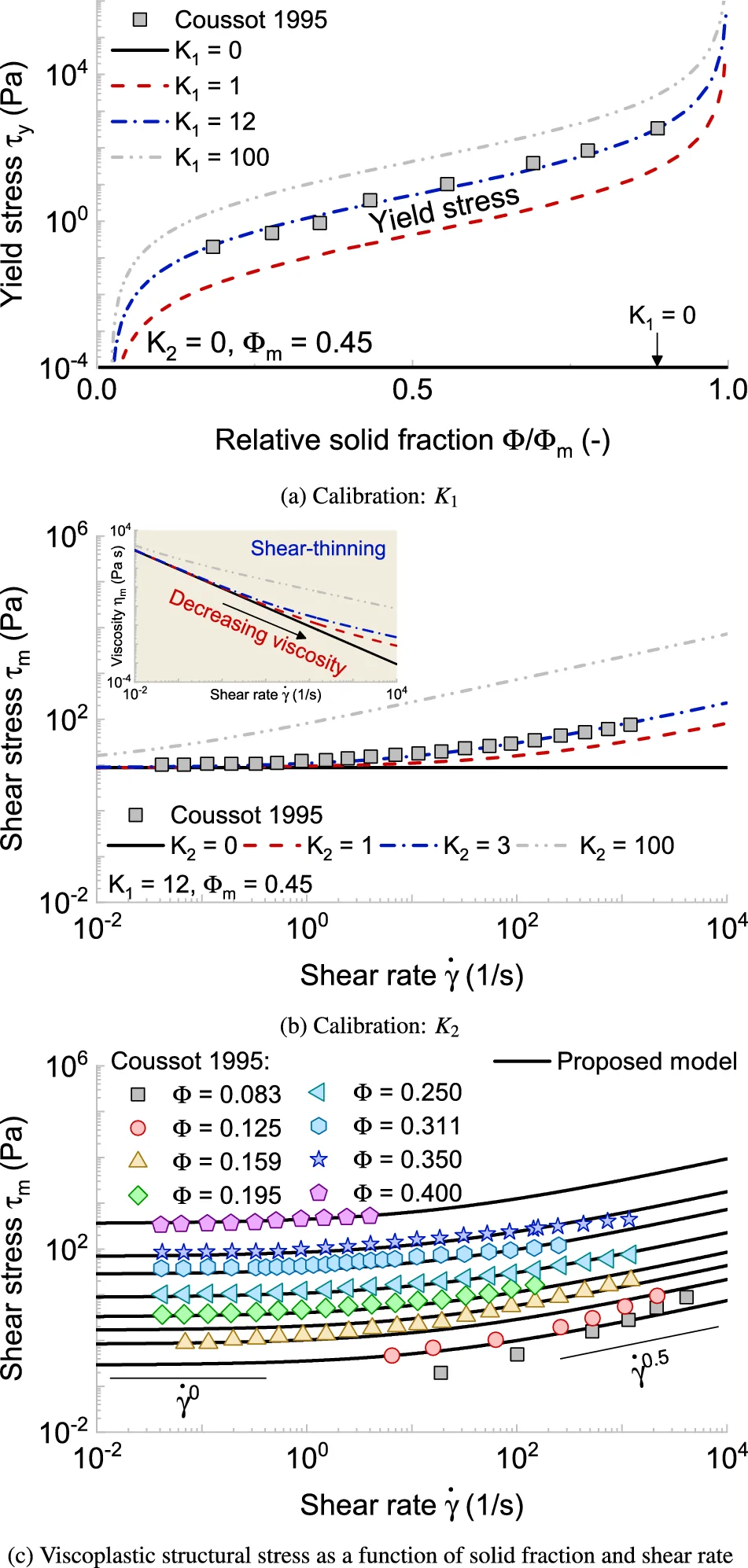
Validation of the viscoplastic structural stress against the data of Coussot [13]

#### Validation of $\boldsymbol{\upsigma }$

Under simple-shear conditions, the equivalent shear stress of the viscoplastic structural stress is obtained as

14 $$\begin{aligned} \begin{aligned} \tau _{\upsigma } = \sqrt{\frac{1}{2} \boldsymbol{\upsigma }_{dev}: \boldsymbol{\upsigma }_{dev}} = \frac{3f_{\Phi }}{\sqrt{2}a} \left( K_1 + K_2\dot{\gamma }^{0.5} \right) \end{aligned} \end{aligned}$$

Equation (14) has the form of a Herschel–Bulkley-type viscoplastic relation. The first term, $3K_1 f_{\Phi }/(\sqrt{2}a)$, represents the residual or yield strength of the viscoplastic structural stress, whereas the second term, $3K_2 f_{\Phi }\dot{\gamma }^{0.5}/(\sqrt{2}a)$, represents the post-yield rate-dependent contribution. The equivalent shear stress of the granular–fluid mixtures can be calculated by $\tau _m= \tau _f + \tau _{\upsigma }$. Equation (13) is therefore used here only as a reduced representation of the viscoplastic structural stress near the onset of flow, including the residual/yield strength and its Herschel–Bulkley-type post-yield extension. It is not intended to replace the full hypoplastic description for general quasi-static deformation, for which Eqs. (6) and (7) remain the appropriate constitutive equations.

The capability of Eq. (13) to represent the residual/yield strength and post-yield viscoplastic structural stress is presented in Fig. 2 using the stress data reported by [13], where the yield stress is evaluated as $\tau _{y} = 3K_1 f_{\Phi }/(\sqrt{2}a)$. This viscoplastic structural-stress tensor is then combined in the following subsection with a suspension stress that accounts for the stress-activated frictional-transition characteristic of fine-granular suspensions.

### Suspension stress

#### Proposed formulation of $\boldsymbol{\Sigma }$

The suspension stress governs the viscous, collisional, and frictional interactions in fine-granular suspensions once flow develops. Unlike the viscoplastic structural stress obtained from the critical-state reduction, the suspension stress is reformulated to capture the stress-activated transition from a lubricated frictionless branch to a frictional branch that underlies discontinuous shear thickening [18]. This is achieved by introducing a frictional-contact state variable and a stress-dependent jamming description, through which repulsion-controlled contact activation is incorporated into the constitutive response [61].

To represent frictional strengthening and dilatancy in granular suspensions, the classical μ(I)-type relations are adopted as the constitutive basis [2, 8],

15 $$\begin{aligned} \begin{aligned} \mu&= \mu _1 + \frac{\mu _2 - \mu _1}{1 + I_0 / I_m}, \qquad \Phi = \frac{\Phi _m}{1 + b I_m} \end{aligned} \end{aligned}$$

where $\mu _1=\tan \phi$ and $\mu _2$ are the lower and upper bounds of the friction coefficient. In the present study, $\mu _2=\mu _1+0.3$ is adopted, consistent with previous work [55]. The constant $I_0=0.279$ denotes the reference inertial number [27]. *b* is a material constant. $I_m$ denotes the modified inertial number, which incorporates viscous and inertial contributions under the combined structural and suspension-stress state and is defined as [55]

16 $$\begin{aligned} \begin{aligned} I_m = d_s \dot{\gamma } \sqrt{\frac{\rho _s}{\upsigma _{vol} + \Sigma _{vol}}}\sqrt{\frac{\alpha _v}{S_t} + \alpha _i} \end{aligned} \end{aligned}$$

where $S_t=\rho _s d_s^2 \dot{\gamma }/\eta _f$ represents the Stokes number. $d_s$ is the mean particle diameter. $\rho _s$ represents the true density of granular materials. $\eta _f$ is the interstitial-fluid viscosity. The parameters $\alpha _v$ and $\alpha _i$ describe the viscous and collisional contributions, respectively. In the present work, $\alpha _v=2$ is fixed, whereas $\alpha _i$ is calibrated for each benchmark case.

However, these relations (15) and (16) do not capture the stress-activated transition underlying discontinuous shear thickening, which is governed by the formation of frictional-contact networks once the repulsive barrier is overcome.

To capture this mechanism, a scalar state variable f∈[0,1] is introduced to represent the fraction of frictional contacts. The activation of *f* is governed by the shear stress associated with the fluid and suspension stresses, so that the transition from a lubricated state to a frictional state is explicitly stress-controlled [6]

17 $$\begin{aligned} f(\tau _{\dot{\gamma }})= \frac{1}{1+\left( \frac{\tau _0}{\tau _{\dot{\gamma }}}\right) ^{3/2}} \end{aligned}$$

where $\tau _0$ is the onset stress. $\tau _{\dot{\gamma }} = \tau _f + \tau _{\Sigma }$ is defined as the shear stress associated with $\textbf{T}_f$ and $\boldsymbol{\Sigma }$ only, excluding the contribution of $\boldsymbol{\upsigma }$.

Using the state variable *f*, the jamming state of the suspension is reformulated as a stress-dependent quantity [61]

18 $$\begin{aligned} \Phi _J = \Phi _{\max } + \left( \Phi _m - \Phi _{\max }\right) f \end{aligned}$$

where $\Phi _{\max }$ is the random close packing volume fraction. The material parameter *b* is also generalised as [6]

19 $$\begin{aligned} b_J = b + \left( b_\infty - b\right) f \end{aligned}$$

where $b_{\infty }$ is a material constant and is set to $b_{\infty }=1.25\,b$ to reduce the number of fitting parameters. Only the parameter *b* needs to be calibrated. Using the second relation in Eq. (15), together with Eqs. (18) and (19), the solid fraction of a fine-granular suspension at steady state is written as

20 $$\begin{aligned} \Phi = \frac{\Phi _J}{1 + b_J I_m} \end{aligned}$$

This relation links the actual solid fraction to the stress-dependent jamming state of the fine-granular suspension. For a given frictional-contact state, this relation can be equivalently inverted for $I_m = \left( \Phi _J-\Phi \right) /\left( b_J\Phi \right)$ in the flowing regime $\Phi <\Phi _J$.

For sufficiently loose states, the volumetric contribution of the viscoplastic structural stress becomes negligible, so that $T_{vol}\approx \Sigma _{vol}$ and, in particular, $\upsigma _{vol}\approx 0$. Combining Eqs. (16) and (20) then gives

21 $$\begin{aligned} \Sigma _{vol} = \rho _s \left( \frac{d_s b_J \Phi }{\Phi _J - \Phi } \right) ^2 \left( \frac{\alpha _v}{S_t} +\alpha _i \right) \dot{\gamma }^2 \end{aligned}$$

Retaining the tensorial structure adopted in the previous constitutive formulation [55], the equivalent shear stress of the suspension stress is written as

22 $$\begin{aligned} \begin{aligned} \tau _{\Sigma } = \sqrt{\frac{1}{2} \boldsymbol{\Sigma }_{dev} : \boldsymbol{\Sigma }_{dev} } = (\mu - \mu _1) \upsigma _{vol} + \mu \eta _2 \dot{\gamma }^2 \end{aligned} \end{aligned}$$

Assuming alignment between the deviatoric suspension stress and the deviatoric strain-rate tensor, one obtains

23 $$\begin{aligned} \frac{\boldsymbol{\Sigma }_{dev}}{\tau _{\Sigma }} = \frac{\dot{\textbf{e}}}{\dot{\gamma } /2} \end{aligned}$$

Consequently, the suspension-stress tensor is written as

24 $$\begin{aligned} \boldsymbol{\Sigma }=\eta _{1}\dot{\textbf{e}}-\eta _{2}\dot{\gamma }^2\textbf{I}\end{aligned}$$

with

25 $$\begin{aligned} \begin{aligned} \eta _{1}&= 2 \frac{\left( \mu - \mu _1 \right) \upsigma _{vol} + \mu \eta _{2}\dot{\gamma }^2}{\dot{\gamma }} \\ \eta _{2}&= \rho _s \left( \frac{d_s b_J \Phi }{\Phi _J - \Phi } \right) ^2 \left( \frac{\alpha _v}{S_t} +\alpha _i \right) \end{aligned} \end{aligned}$$

The coefficient $\eta _{2}$ is evaluated from the above expression when $\Phi <\Phi _J$, whereas $\eta _{2}=0$ is imposed for $\Phi \ge \Phi _J$, so that the deformation in this regime is interpreted as being dominated by the viscoplastic structural stress $\boldsymbol{\upsigma }$ rather than by the suspension-stress contribution. Unlike Eq. (8), Eq. (24) incorporates the stress-activated viscosity change associated with overcoming the short-range repulsive barrier through the dependence of $\eta _{1}$ and $\eta _{2}$ on the frictional-contact state. The coefficients $\eta _1$ and $\eta _2$ describe the deviatoric and isotropic contributions of the same suspension stress and are constitutively coupled rather than physically independent.

When the stress dependence of the jamming state is suppressed, i.e. by replacing $\Phi _J$ and $b_J$ with their stress-independent counterparts $\Phi _m$ and *b*, respectively, Eqs. (24) and (25) reduce to the non-repulsive form, consistent with the reference model presented by Wang and Wu for coarse granular–fluid mixtures [55]. In this limiting case, the repulsion-controlled frictional transition is suppressed, and DST is therefore not activated.

**Fig. 3:**
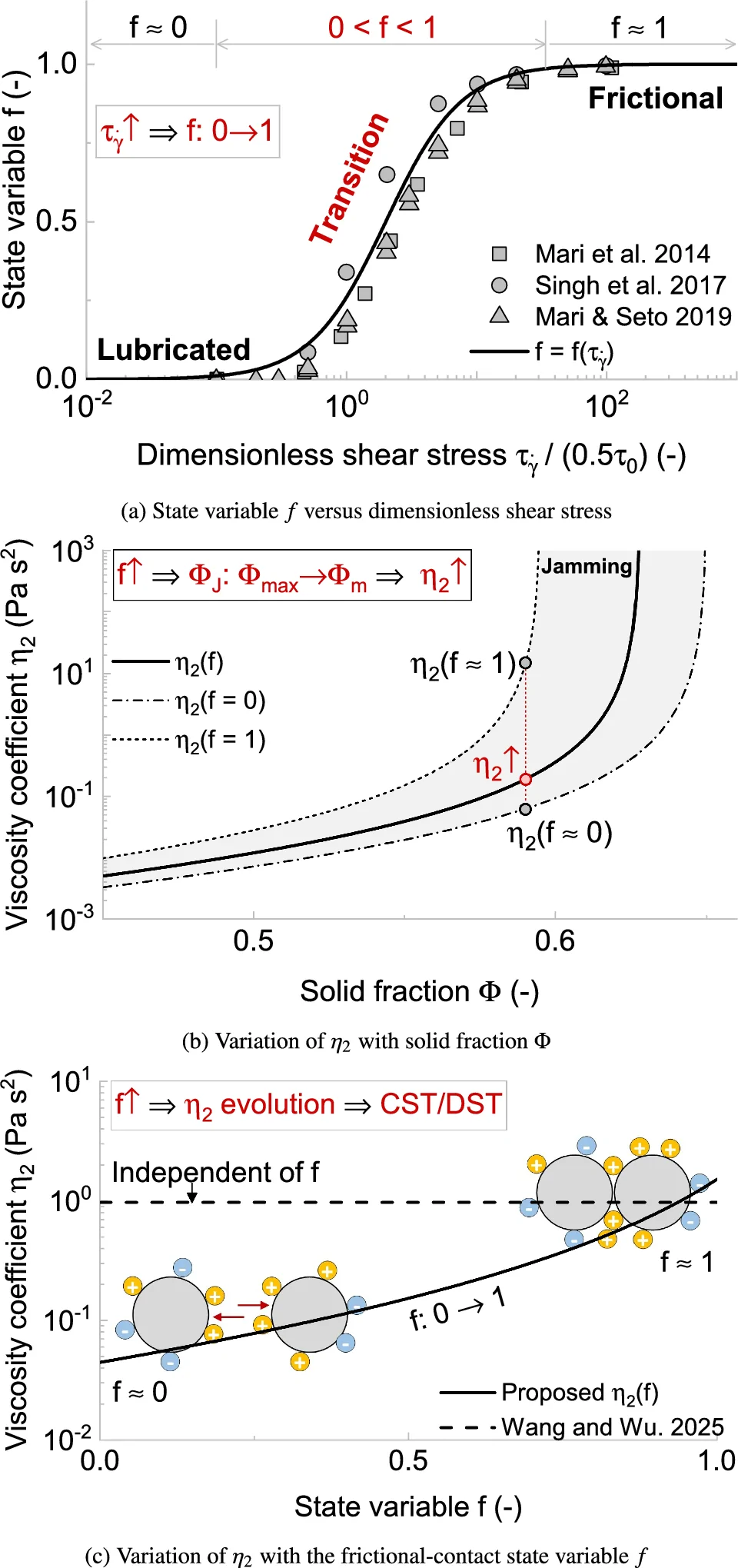
Effect of the frictional-contact state variable and solid fraction on the volumetric coefficient coefficient

Figure 3 illustrates the stress-activated frictional transition and the role of the stress-dependent jamming state in the suspension resistance. For a direct comparison that isolates the effect of the present stress-dependent formulation, the calculations use the same parameter values as the non-repulsive formulation of Wang and Wu [55]: $\Phi _m=0.595$, $\Phi _{\max }=0.65$, b=0.57, $\eta _f=0.001~\mathrm {Pa\cdot s}$, $\alpha _i=0$, and μ=1. The frictional-contact state variable $f=f(\tau _{\dot{\gamma }})$ is compared with experimental data from previous studies [31, 32, 47]. The constitutive evolution can be summarised as follows:

- **Frictional-contact activation.** As the applied suspension stress increases, *f* evolves from 0 to 1, describing the progressive transition from lubricated to frictional contacts.
- **Stress-dependent jamming.** The increase in *f* shifts the jamming state from $\Phi _{\max }$ towards $\Phi _m$, $\Phi _J=\Phi _{\max }+(\Phi _m-\Phi _{\max })f,$ while $b_J=b+(b_\infty -b)f$ evolves in the opposite direction. At fixed Φ, the resulting decrease in the distance to jamming, $\Phi _J-\Phi$, strongly increases the suspension resistance through the dependence of $\eta _2$ on $(\Phi _J-\Phi )^{-2}$. Thus, $\Phi _J$ provides the constitutive link between frictional-contact activation and the increase in suspension resistance. The coefficient $b_J=b+(b_\infty -b)f$ evolves simultaneously with *f* and further modulates this resistance evolution.
- **Macroscopic thickening.** The increasing suspension resistance drives the response from the lubricated branch through the CST/DST transition towards the frictional branch. When the stress dependence of $\Phi _J$ and $b_J$ is suppressed, $\eta _2$ becomes independent of *f* and the repulsion-controlled thickening mechanism is lost.

The stress-dependent jamming state therefore governs the transition between the lubricated and frictional suspension branches, while the viscoplastic structural stress remains responsible for low-rate yielding and apparent shear thinning.

**Fig. 4:**
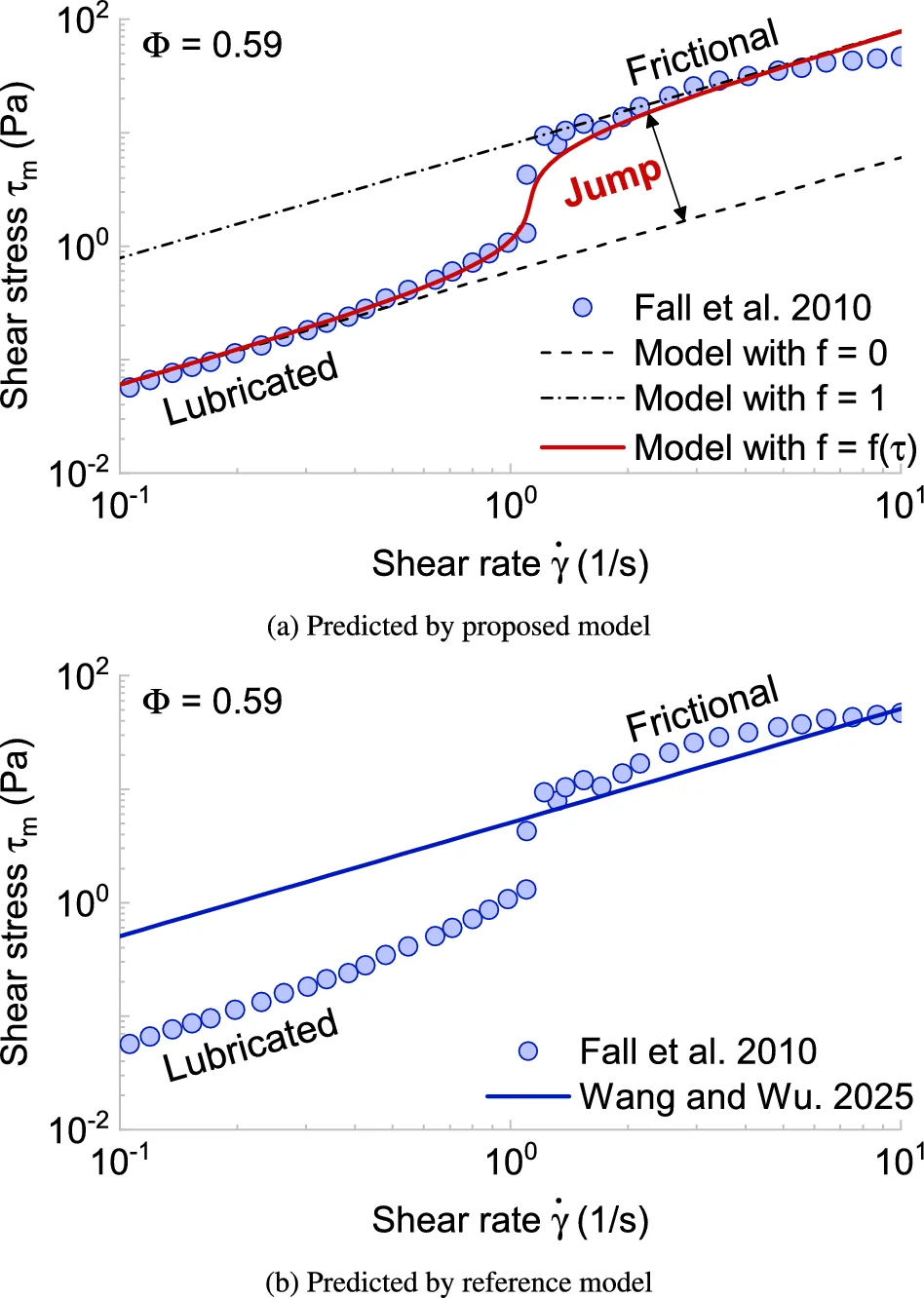
Shear stress response across DST transition

#### Validation of $\boldsymbol{\Sigma }$

The proposed formulation in Eq. (24) is assessed using the experimental data reported by [18], for which the viscoplastic structural stress and inertial contributions can be considered negligible; hence, the response is governed primarily by the viscous and frictional components of the fine-granular suspension. In the present study, unless otherwise specified, all fine-granular suspension cases are based on the material parameters reported by [18], namely $d_s=40~\mu \textrm{m}$, $\rho _s=1050~\mathrm {kg/m^3}$, and $\eta _f=0.001~\mathrm {Pa\cdot s}$. The internal friction angle is taken as $\phi = 20.8^\circ$, consistent with the value proposed by [8]. The remaining constitutive parameters are case-dependent and are assigned the following values for this case: $\Phi _{\max }=0.605$, $\Phi _m=0.595$, b=0.68, and $\tau _0=1.8~\textrm{Pa}$.

Figure 4 shows the resulting flow curves together with the evolution of the state variable *f*, with the rheological model presented by [55] used as the reference model. The equivalent shear stress of the granular–fluid mixtures is calculated by $\tau _m= \tau _f + \tau _{\Sigma }$. As shown in Fig. 4, the response can be divided into three states: (i) at low shear rates, it remains close to the f≈0 branch, exhibiting a macro-viscous, frictionless behaviour; (ii) with increasing stress, a rapid transition occurs over a narrow shear-rate range, during which *f* sharply increases from near 0 to 1, accompanied by a pronounced stress rise corresponding to discontinuous shear thickening; and (iii) at higher shear rates, the response approaches the fully frictional branch (f≈1), characterised by a significantly enhanced flow resistance due to the development of a frictional-contact network. The evolution of *f* confirms that the macroscopic stress jump is directly associated with the rapid activation of frictional contacts within the suspension. In contrast, when DST is not accounted for, the reference model fails to reproduce the stress jump behaviour, as shown in Fig. 4b.

### Remark

The proposed formulation decomposes the effective granular stress into a viscoplastic structural stress and a suspension stress. The former, given by Eq. (13), captures the low-rate yielding and apparent shear-thinning branch, whereas the latter, defined by Eqs. (24), accounts for the viscous, collisional, and repulsion-controlled frictional-contact contributions responsible for CST, DST, and jamming.

The observed rheological transitions are interpreted as changes in the relative contributions of the structural and suspension stresses within the effective granular stress, rather than as switches between separate constitutive laws. This provides a unified mechanical interpretation of the transition from solid-like yielding to suspension flow and frictional thickening.

By combining these two stress contributions within the effective-stress framework of Eqs. (1)–(3), the present model describes the transition from flow onset and structural shear thinning to macro-viscous flow, stress-activated thickening, and frictional flow or jamming.

## Model performance and parameter analysis

All calculations in the following sections are performed using the complete constitutive framework defined by Eqs. (1)–(3). For clarity, under homogeneous simple shear, the resulting equivalent shear stress $\tau _m=\sqrt{\frac{1}{2}\textbf{T}_{m,dev}:\textbf{T}_{m,dev}}$ can be written in the following scalar form

26 $$\begin{aligned} \begin{aligned} \tau _m =&\underbrace{\eta _f\left( 1 + \frac{5}{2} \Phi \right) \dot{\gamma }+ (\mu - \mu _1) \upsigma _{vol} + \mu \eta _2 \dot{\gamma }^2}_{\tau _{\dot{\gamma }}} \\&+ \frac{3f_{\Phi }}{\sqrt{2}a} \left( K_1 + K_2\dot{\gamma }^{0.5} \right) \end{aligned} \end{aligned}$$

where $\tau _{\dot{\gamma }}$ collects the fluid and suspension-stress contributions, while the last term denotes the viscoplastic structural-stress contribution. The relative viscosity of the granular–fluid mixture is defined as $\eta _m^*=\eta _m/\eta _f$.

**Fig. 5:**
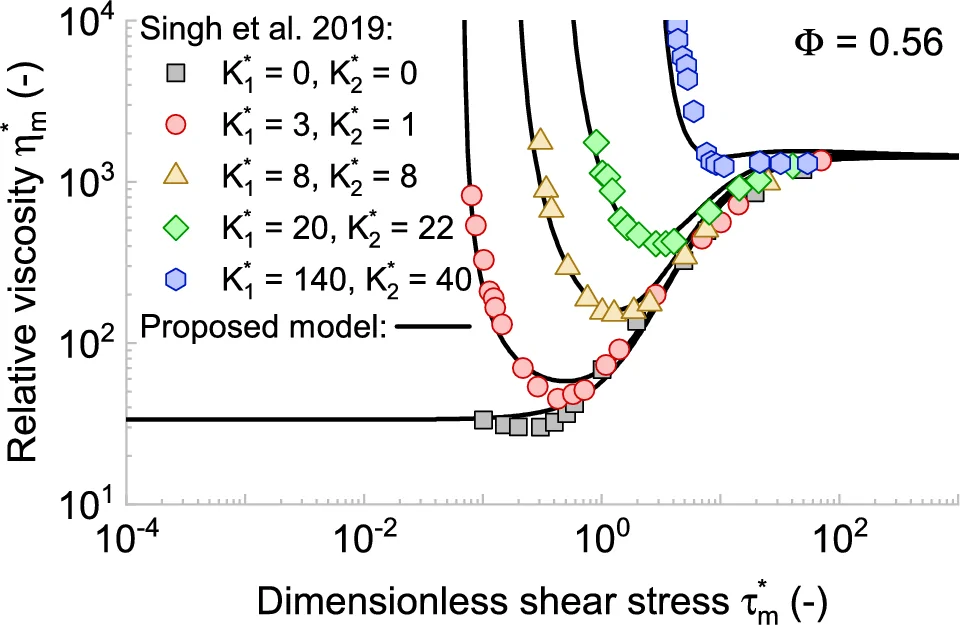
Steady-state flow curves at a solid fraction of Φ=0.56 for different values of the yield stress

**Fig. 6:**
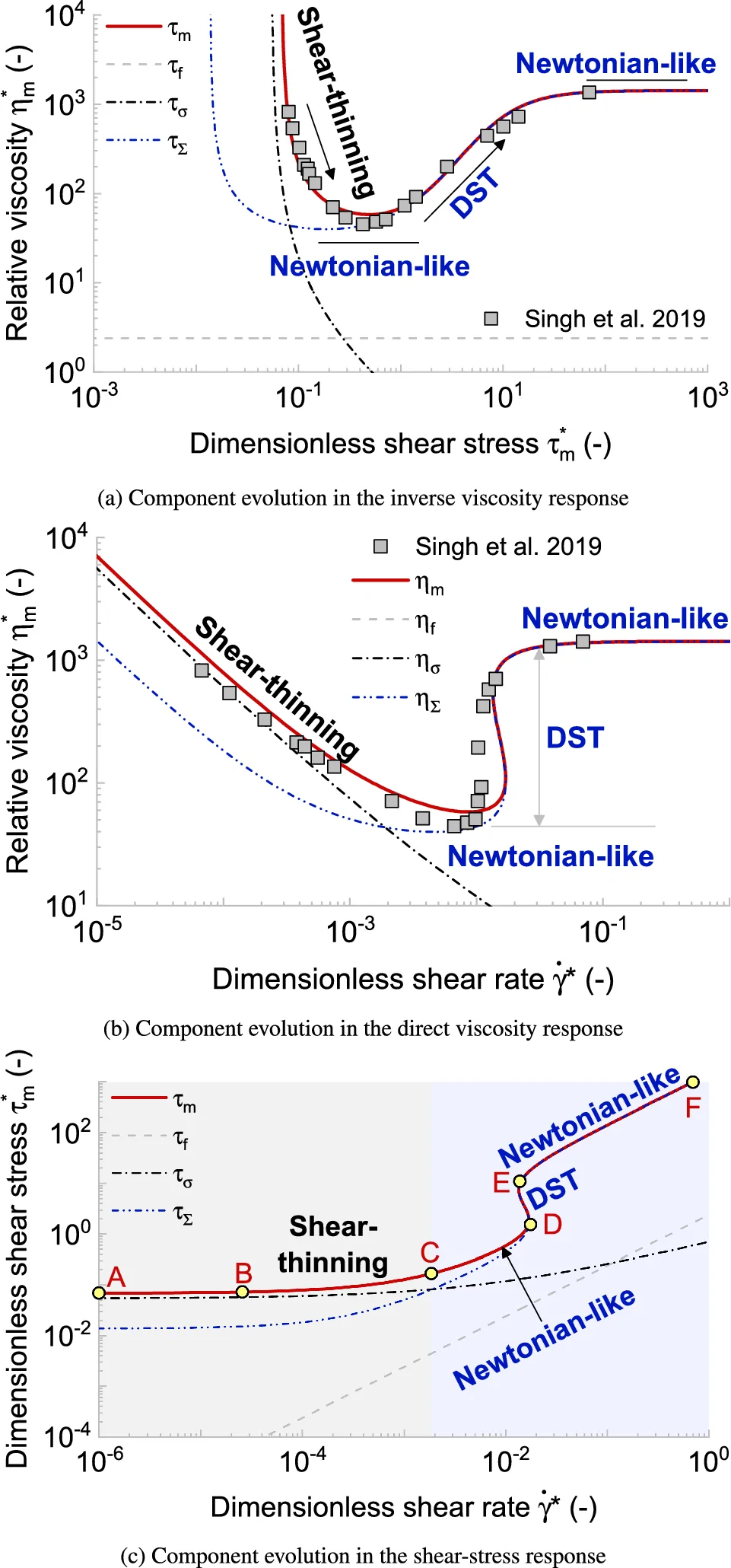
Predicted flow response and evolution of stress contributions for the cohesive frictional suspension reported by Singh et al. [49]

**Fig. 7:**
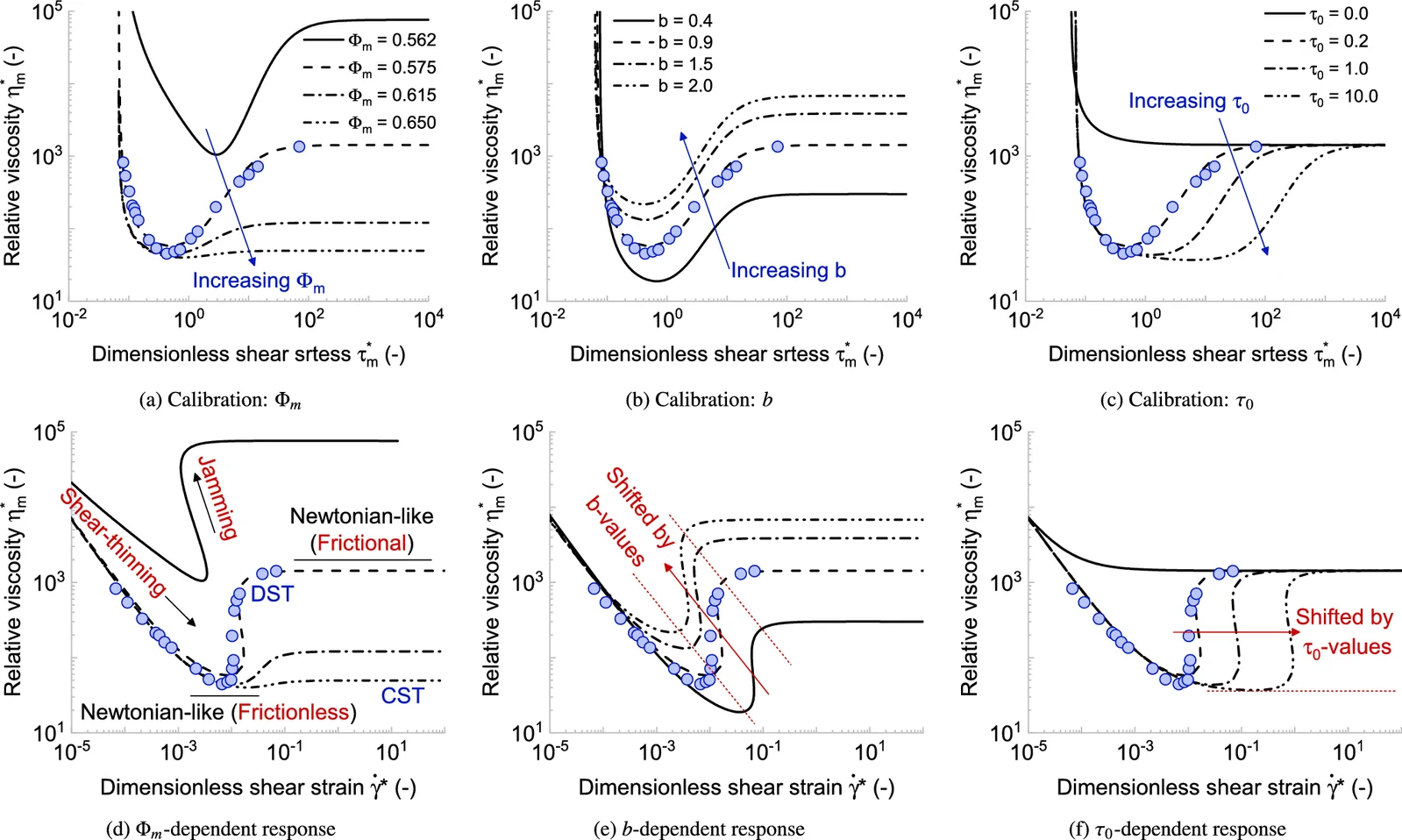
Calibration and parametric effects of $\Phi _m$, *b*, and $\tau _0$ on the predicted flow response

The dataset reported by Singh et al. [49] is selected because it provides a representative cohesive frictional suspension in which yielding, shear thinning, stress-activated thickening, and shear jamming occur within a single material system. The data correspond to a fixed solid fraction of Φ=0.56, with five levels of viscoplastic structural stress considered, making them suitable for assessing the influence of $\boldsymbol{\upsigma }$ on the full flow transition. Since the original study did not specify a reference shear rate for normalisation, $\dot{\gamma }_0=100~\mathrm {s^{-1}}$ is adopted here, and the values of $K_1$ and $K_2$ are obtained by inversion based on this reference scale. In Fig. 5, $K_1^*=K_1/10^{-4}$ and $K_2^*=K_2/10^{-5}$ are calibrated according to the procedure outlined in Sect. 3.2.2. The dimensionless shear stress is defined as $\tau _m^*=\tau _m/(\eta _f\dot{\gamma }_0)$, and the dimensionless shear rate is given by $\dot{\gamma }^*=\dot{\gamma }/\dot{\gamma }_0$. The remaining material parameters are set as $\Phi _m=0.575$, $\Phi _{\max }=0.65$, b=0.9, $\tau _0=0.2$ Pa, and $\alpha _i = 0$.

As shown in Fig. 5, the proposed model shows good agreement with the flow curves reported by [49]. To further clarify the underlying mechanism, one representative flow curve (with $K_1^* = 3$ and $K_2^* = 1$) in Fig. 5 is selected for detailed stress decomposition. Figure 6 shows the evolution of the total shear stress $\tau _m$, the fluid stress $\tau _f$, the viscoplastic structural stress $\tau _{\upsigma }$, and the stress-activated suspension stress $\tau _{\Sigma }$, indicating that the mixture undergoes a sequence of distinct deformation regimes:

- **A–B: quasi-static onset.** The flow starts from a finite structural backbone strength. The total shear stress is dominated by the residual component, $\tau _m \simeq \tau _{\upsigma }$, with $\partial \tau _m/\partial \dot{\gamma } \approx 0$.
- **B–C: apparent shear-thinning branch.** After yielding, the viscoplastic structural contribution follows the Herschel–Bulkley-type scaling in Eq. (14). Its viscosity contribution decreases with increasing shear rate, owing to the $\dot{\gamma }^{-1}$ residual/yield term and the $\dot{\gamma }^{-1/2}$ post-yield term.
- **C–D: macro-viscous, lubricated branch.** The suspension stress becomes dominant, while direct frictional contacts remain largely inactive, f≈0. The response is Newtonian-like, $\tau _m \simeq \tau _f+\tau _{\Sigma }\propto \dot{\gamma }$.
- **D–E: stress-activated thickening transition.** When the applied stress exceeds the repulsive barrier, frictional contacts are activated, f increases from 0 to 1. The jamming state shifts from the frictionless to the frictional branch, producing a DST-type jump and a non-monotonic flow segment with $\partial \dot{\gamma }/\partial \tau _m<0$.
- **E–F: macro-viscous, frictional branch.** After the transition, the suspension flows on the fully frictional branch, f≈1. The response again becomes Newtonian-like but with a much larger resistance, $\tau _m\propto \dot{\gamma }$, controlled by the frictional-contact network.

Therefore, the proposed framework captures the transition from quasi-static yielding and structural shear thinning to macro-viscous flow, stress-activated DST, and the final frictional flow branch within a single constitutive description.

The basic effects of the solid fraction Φ and fluid viscosity $\eta _f$ have been discussed in a related granular–fluid framework and are not revisited here in detail [55]. The parametric influence of $\Phi _m$, *b*, and $\tau _0$ is further presented in Fig. 7. These parameters control different aspects of the DST response and can therefore be identified in a physically interpretable manner. When $\Phi _m$ approaches $\Phi _{\max }$, the contrast between the frictionless and frictional jamming states becomes weak, so that the discontinuous transition is largely suppressed and the response approaches continuous shear thickening. Decreasing $\Phi _m$ brings the suspension closer to the frictional jamming limit, thereby sharpening the transition and, in the inverse representation, potentially leading to a near-vertical branch or loss of monotonicity. In addition, $\Phi _m$ has a pronounced influence on the entire deformation process, affecting not only the thickening transition but also the overall evolution from the low-rate branch to the frictional branch. By contrast, the parameter *b* controls the sensitivity of the jamming relation to the modified inertial number. Increasing *b* raises the overall viscosity level and modifies the steepness of the transition between the frictionless and frictional branches. The onset stress $\tau _0$ mainly shifts the stress scale at which the frictional transition is activated, and is therefore particularly important for locating the DST region. Accordingly, $\Phi _m$ is treated as the primary parameter to be calibrated first, whereas *b* and $\tau _0$ are subsequently obtained through inversion.

## Model validation

**Table 1:** Model parameters used in the four benchmark cases

Parameter	Symbol	Unit	Case I	Case II	Case III	Case IV
Maximum solid fraction	$\Phi _{\max }$	–	0.7	0.65	0.64	0.64
Jamming volume fraction	$\Phi _m$	–	0.515	0.595	0.533	0.577
Constitutive constant	$K_1$	Pa	0.08	0.0	0.02	0.0
Constitutive constant	$K_2$	Pa	0.0	0.0	0.02	0.0
Constitutive constant	*b*	–	7.0	0.57	0.8	0.53
Inertial weighting factor	$\alpha _i$	–	1.0	0.0	0.0	0.8
Onset stress	$\tau _0$	Pa	-	0.2	0.2	0.01–$10^6$

**Fig. 8:**
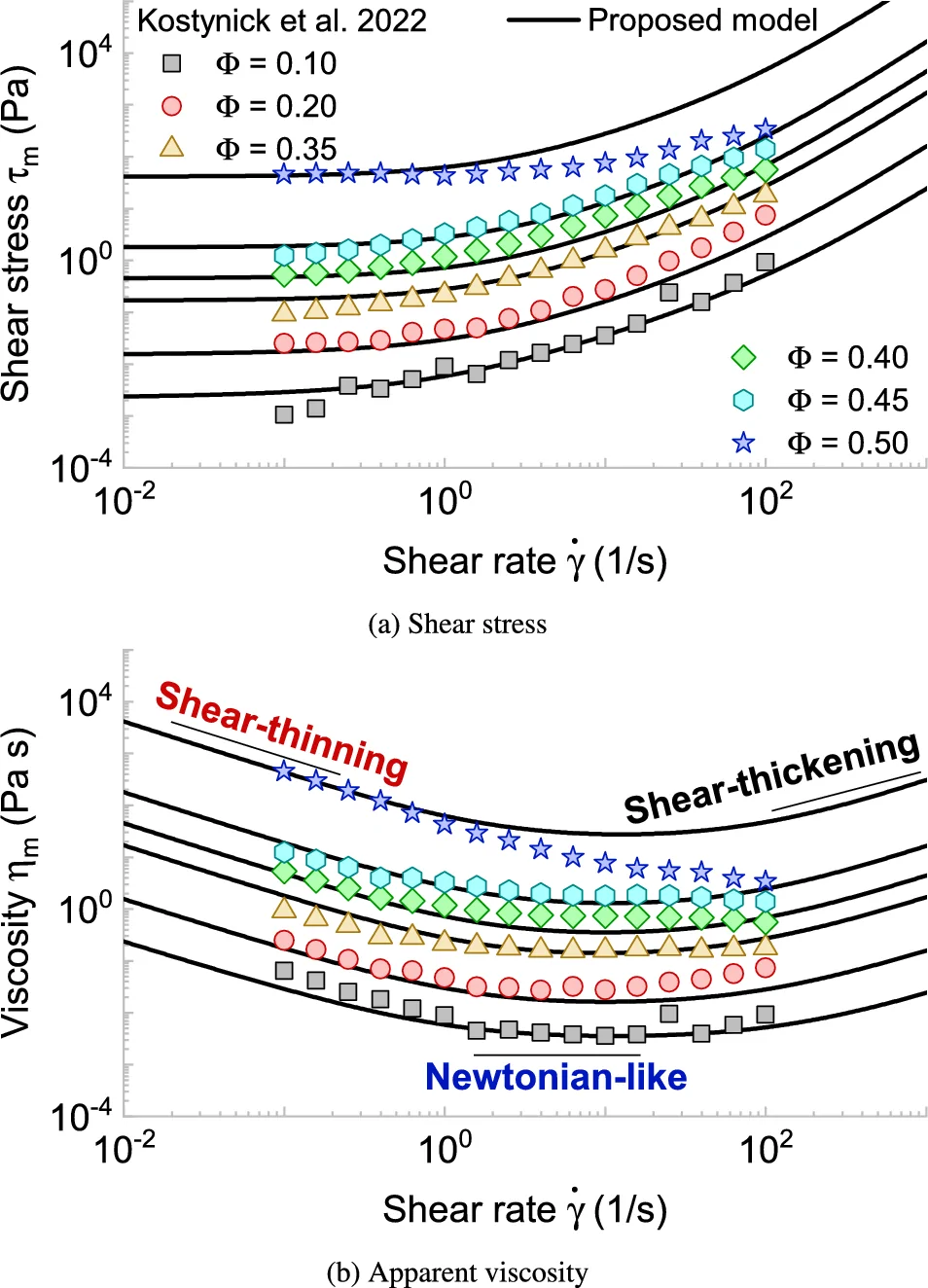
Measured and predicted response of debris-flow materials under homogeneous shear [28]

**Fig. 9:**
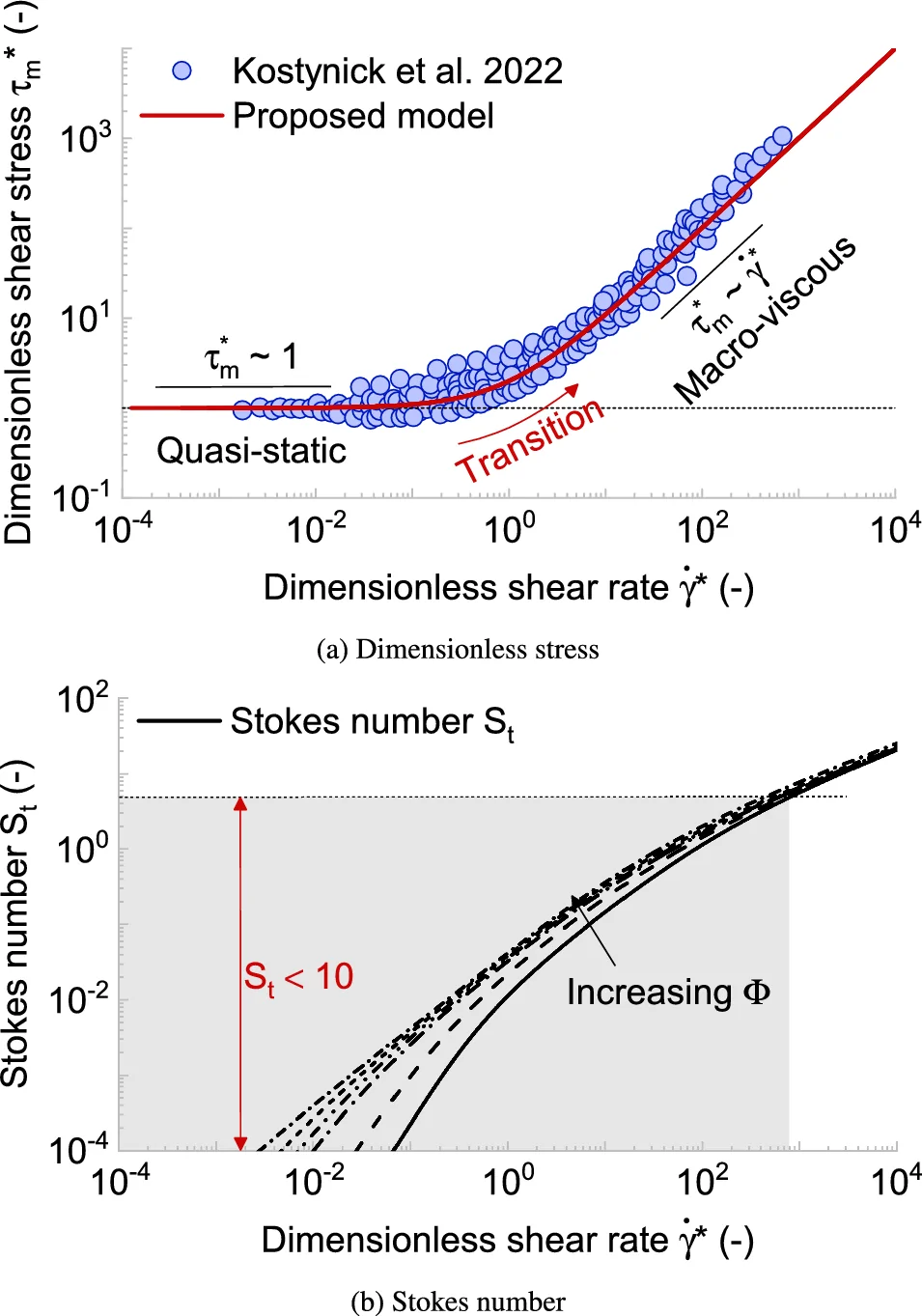
Transition from quasi-static to macro-viscous behaviour in debris-flow materials

**Fig. 10:**
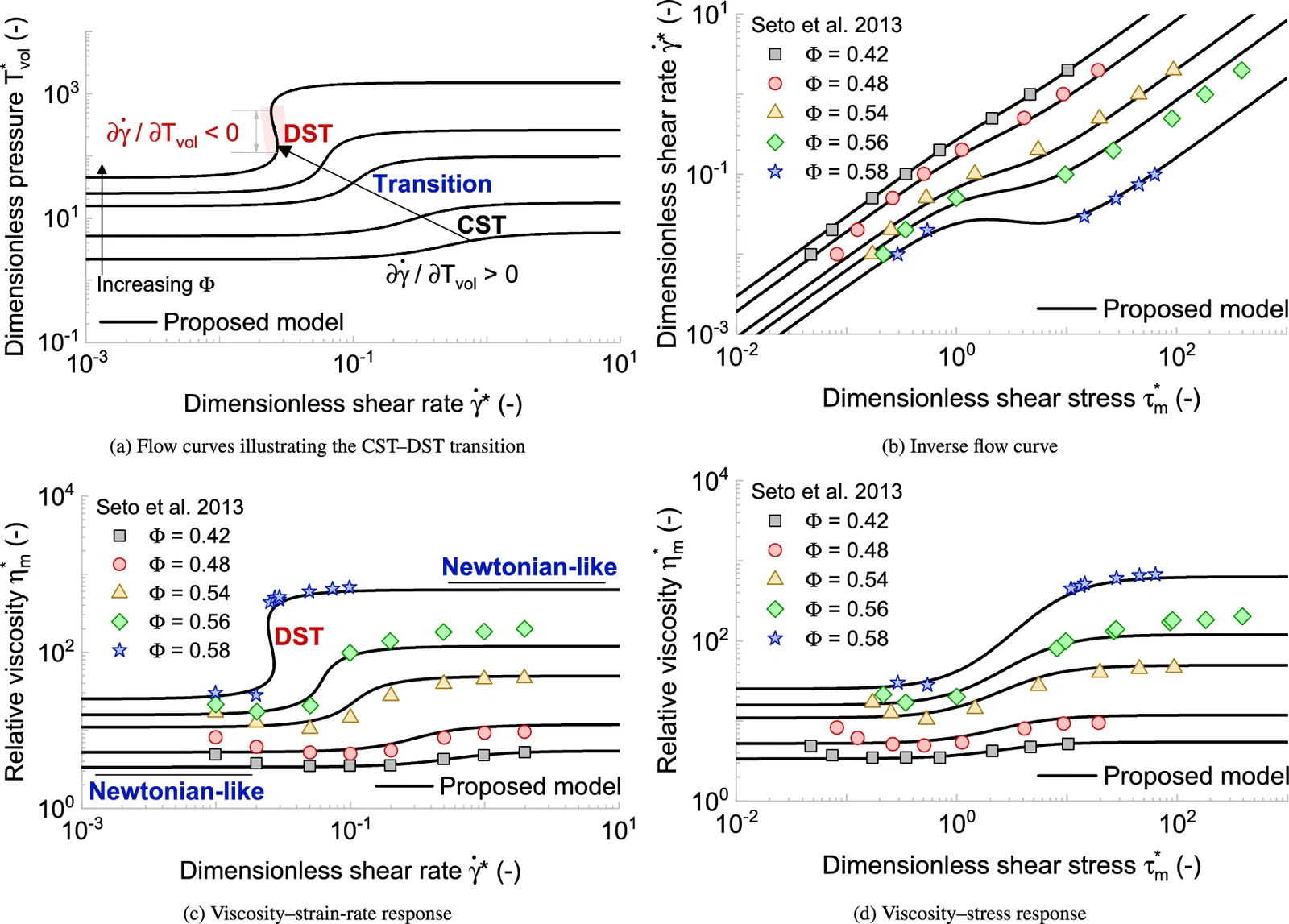
Model predictions for DST within the macro-viscous regime [44]

**Fig. 11:**
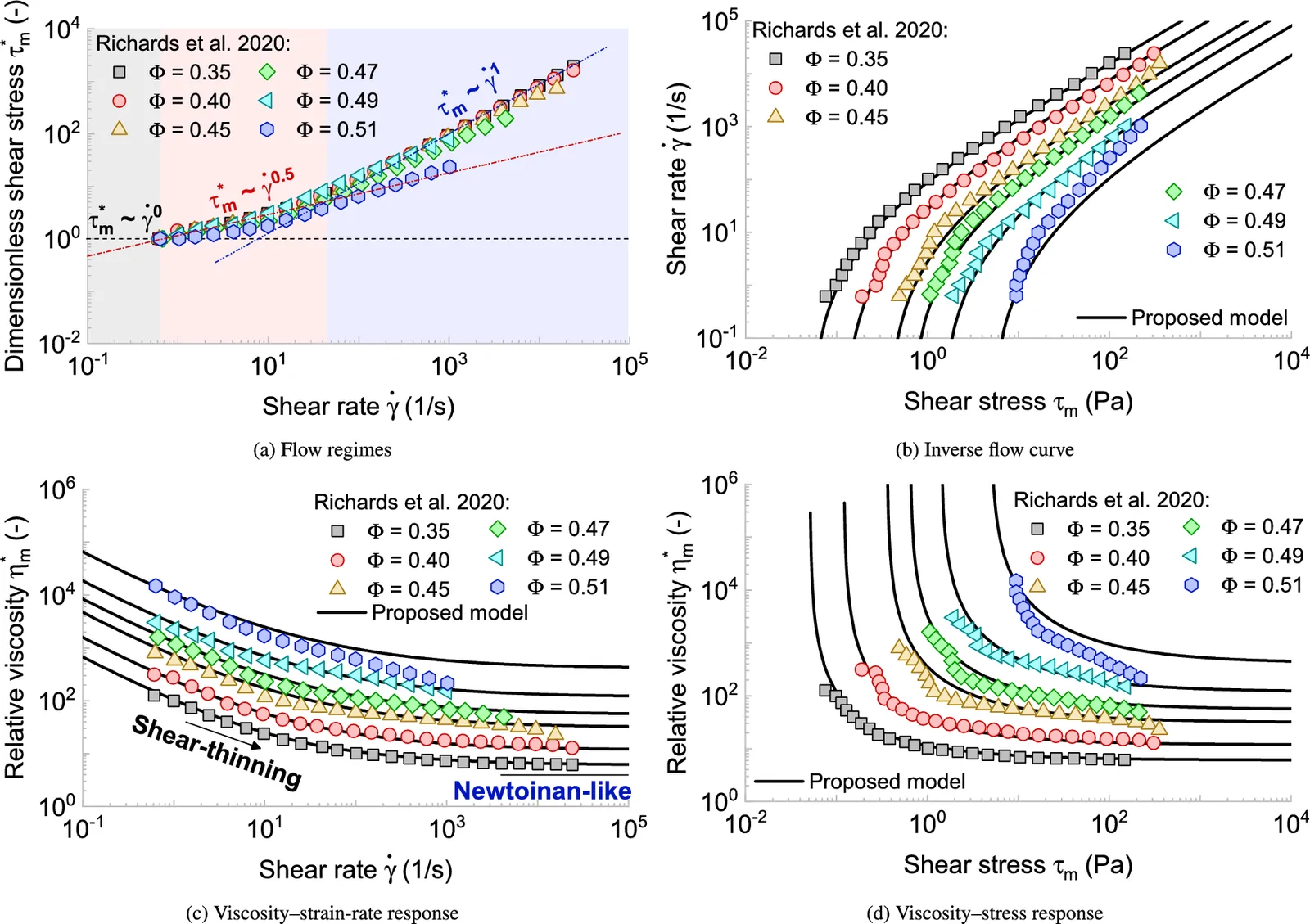
Model predictions for suspensions with finite residual strength at flow onset [41]

**Fig. 12:**
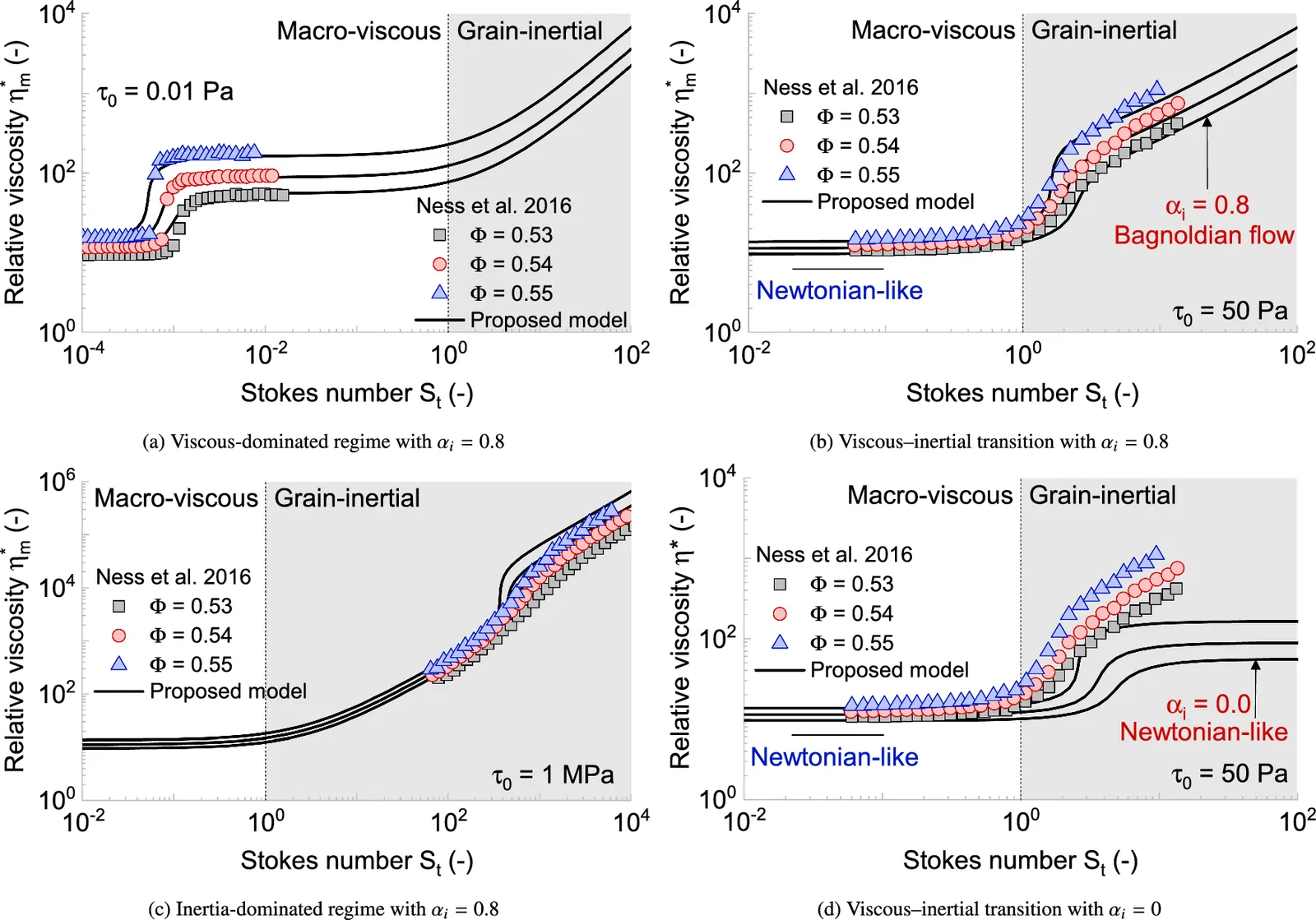
Model predictions for DST across macro-viscous, transitional, and grain-inertial regimes [34]

As discussed in Sect. 2, the constitutive framework is evaluated under homogeneous simple shear at fixed volume, which provides a canonical setting for isolating the local rheological response of granular–fluid mixtures. All benchmark cases in this section are computed within this configuration, so that the respective roles of viscoplastic structural resistance, suspension-flow resistance, and stress-activated frictional transitions can be examined in a controlled manner.

All four benchmark cases are described using the same constitutive formulation, with different rheological responses obtained through the corresponding material parameters. The cases were selected to examine complementary aspects of the proposed framework under distinct rheological conditions:

- Case I examines the non-DST transition from quasi-static to macro-viscous flow;
- Case II considers classical DST within the macro-viscous regime;
- Case III assesses the influence of finite structural resistance at flow onset and its modification of the DST response;
- Case IV examines the interaction between frictional thickening and particle inertia across the macro-viscous and grain-inertial regimes.

Together, these cases test the roles of structural resistance, stress-activated frictional thickening, and particle inertia within the same constitutive formulation, demonstrating its ability to describe distinct granular–fluid rheological responses without changing the underlying constitutive structure. The parameter values listed in Table 1 are taken directly from the cited studies when explicitly reported; otherwise, they are inferred following the calibration procedures described in Sects. 3.2.2 and 3.3.2.

### Case I: Debris-flow rheology without DST

To assess the generality of the proposed framework in the absence of DST, the model is compared with the experimental data reported by Kostynick et al. for natural debris-flow materials [28]. In these experiments, the flow behaviour is governed by frictional contacts and viscous interactions, while repulsion-controlled frictional activation leading to DST is not evident.

Within the present framework, this situation corresponds to a regime in which the stress-activated frictional-transition mechanism introduced in Sect. 3 does not play a dominant role, and the response is governed by the interplay between the viscoplastic structural stress and the suspension stress. The data therefore provide a suitable benchmark for examining the transition from quasi-static to macro-viscous behaviour without invoking DST.

Figure 8 shows the comparison between the model predictions and the experimental measurements. As shown in Fig. 8a, the shear stress increases monotonically with shear rate for all solid fractions, without exhibiting the abrupt stress jump characteristic of DST. The corresponding apparent viscosity shown in Fig. 8b displays a pronounced shear-thinning behaviour at low shear rates, followed by a gradual approach towards a macro-viscous regime at higher shear rates. This trend reflects the progressive reduction of the relative contribution of the viscoplastic structural stress as the suspension-flow resistance becomes dominant.

The transition is further presented in Fig. 9, where the dimensionless shear stress and shear rate are defined as $\tau _m^* = \tau _m / \tau _y$ and $\dot{\gamma }^* = \eta _m \dot{\gamma }/ \tau _y$, respectively. At low shear rates, the dimensionless stress remains close to a constant level, indicating a quasi-static regime controlled by the residual granular strength. As the shear rate increases, the response departs from this plateau and transitions towards a macro-viscous regime characterised by an approximately linear scaling with shear rate. The evolution of the Stokes number shown in Fig. 9b indicates that the entire experimental range remains within $S_t<10$, while most of the data lie in the regime $S_t<1$. This implies that the observed transition occurs predominantly within the frictional–viscous domain, i.e. from quasi-static to macro-viscous behaviour, rather than towards an inertia-dominated regime.

This benchmark demonstrates that the proposed constitutive framework is able to reproduce the continuous transition from quasi-static to macro-viscous behaviour in fine-granular materials, including the shear-thinning response at low shear rates, without requiring the activation of discontinuous shear thickening. This provides further support for the consistency of the model across different rheological regimes.

### Case II: Discontinuous shear thickening in the macro-viscous regime

This case considers discontinuous shear thickening in the absence of a significant viscoplastic structural contribution at flow onset. It corresponds to suspensions in which the residual granular strength is negligible compared to the suspension stress, so that the response is effectively governed by the viscous and frictional components. The purpose of this benchmark is to assess whether the proposed model can reproduce the classical frictionless-to-frictional transition within the macro-viscous regime. The comparison is made with the data of [44].

The results are presented in terms of the dimensionless shear stress $\tau ^*=\tau _m/(\eta _f\dot{\gamma }_0)$ and dimensionless shear rate $\dot{\gamma }^*=\dot{\gamma }/\dot{\gamma }_0$, where $\dot{\gamma }_0=100$ 1/*s* is introduced as a reference scaling parameter. As shown in Fig. 10, the model reproduces the essential features of macro-viscous DST. At low shear rates, the response follows a frictionless branch with weak rate dependence. As the stress increases, a transition region develops, connecting the low-viscosity frictionless branch to a high-viscosity frictional branch. With increasing Φ, this transition becomes progressively sharper and eventually leads to a discontinuous stress jump, in agreement with the reported experimental trends.

The inverse flow curves further indicate that, at sufficiently high Φ, the constitutive response loses monotonicity, reflecting the proximity to the frictional jamming state. These results demonstrate that the proposed framework is able to reproduce classical macro-viscous DST as a stress-activated transition within the suspension-stress contribution, consistent with the limiting behaviour illustrated in Sect. 3.3.2.

### Case III: Stress-activated response with finite structural resistance

This case addresses suspensions in which a finite viscoplastic structural stress is present at flow onset for different solid fractions. Such behaviour is relevant for dense fine-granular suspensions encountered in geotechnical contexts, where the material may not start from a purely viscous state. The comparison is made with the data of Richards et al.[41].

The model predictions in Fig. 11 indicate that, at low shear rates, the stress is dominated by a finite viscoplastic structural contribution, giving rise to a plateau at flow onset. The dimensionless shear stress is defined as $\tau ^*_m = \tau _m / \tau _y$. As the shear rate increases, the relative contribution of the viscoplastic structural stress decreases and the response transitions towards a macro-viscous regime, accompanied by a monotonic decrease in relative viscosity. Although the flow curve steepens at higher stresses, no distinct shear-thickening regime emerges in the viscosity–shear-rate representation. This behaviour is consistent with the framework of Richards et al. [41], in which frictional contacts are active from the onset of flow in the presence of a finite yield stress, while adhesive constraints are progressively relaxed. In contrast to classical DST, which arises from a stress-induced transition between lubricated and frictional states, such a transition is absent here. The resulting response is therefore governed by yielding and structural relaxation over an extended stress range, precluding the abrupt viscosity increase characteristic of DST.

These results indicate that yielding and shear thickening are governed by competing stress scales rather than independent mechanisms. The presence of a finite yield stress imposes a dominant threshold that controls both flow initiation and microstructural evolution, thereby delaying or suppressing the emergence of DST (see a similar trend in Fig. 5). The absence of a distinct shear-thickening regime thus reflects the predominance of yielding over stress-activated contact transitions in this class of suspensions.

### Case IV: Discontinuous shear thickening across viscous–inertial regimes

The final case examines the influence of particle inertia on the DST response and its interaction with the stress-activated frictional transition. The objective is to demonstrate that the proposed framework can describe discontinuous shear thickening across macro-viscous, inertia-influenced, and transitional regimes within a single constitutive formulation.

The comparison is made with the data of [34]. In this case, the inertial contribution is introduced through the parameter $\alpha _i$, which controls the relative importance of collisional effects in the modified inertial number. Different regimes are explored by varying the stress level and the inertial weighting factor.

As shown in Fig. 12, the model captures the main classes of behaviour observed in the benchmark data. When inertial effects remain weak at the transition point, the response retains the macro-viscous structure observed in Sect. 4. As the inertial contribution increases, the high-stress branch progressively departs from viscous scaling and approaches a grain-inertial regime. In the limiting case, the DST transition occurs directly within the inertia-dominated regime. Intermediate cases exhibit a smooth crossover in which frictional activation and viscous–inertial transition occur over overlapping ranges of stress and shear rate.

These results indicate that the proposed model provides a unified description of DST across different flow regimes, without requiring separate constitutive laws for viscous and inertial conditions. The transition remains governed by the stress-activated evolution of the frictional state variable *f*, while the asymptotic behaviour of the high-stress branch is controlled by the balance between viscous and inertial contributions.

## Depth-averaged long-wave analysis in a macro-viscous limit

This section presents a minimal free-surface application of the proposed constitutive framework, focusing on whether DST-induced non-monotonicity survives depth averaging and can trigger shallow-flow instability [16]. More complete boundary-value problems, such as front propagation and constant-speed travelling states [7], require the full coupled formulation and are beyond the scope of the present work.

The analysis is restricted to a macro-viscous limiting case, using the material parameters of Case II in Sect. 5.2. The flow is assumed to be shallow, slowly varying, and inertia-free in the momentum balance. The solid fraction Φ is taken as spatially uniform and constant in time, capillary effects are neglected, the pressure field is hydrostatic, and no-slip and traction-free conditions are imposed at the base and free surface, respectively. Under these assumptions, the derivation provides a constitutive prototype for how the proposed framework may enter a free-surface boundary-value problem. The detailed derivation is given in Appendix B; only the main framework is presented here.

When $\Phi <\Phi _J(\tau )$, the local macro-viscous constitutive closure may then be written in the compact form

27 $$\begin{aligned} \dot{\gamma } = \Gamma (\tau ;\Phi ) := \frac{\tau }{ \eta _f \left[ 1+\dfrac{5}{2}\Phi + \alpha _v\,\mu (\tau ) \left( \dfrac{b_J(\tau )\Phi }{\Phi _J(\tau )-\Phi } \right) ^2 \right] } \end{aligned}$$

where τ denotes the local scalar shear stress. Since $\boldsymbol{\upsigma }=\textbf{0}$ in the macro-viscous limiting case, $\tau =\tau _m=\tau _f+\tau _{\Sigma }$. Under the present unidirectional shallow-flow approximation, this scalar stress is identified with the shear component $\tau _{xz}$ of the mixture stress.

We consider a two-dimensional shallow free-surface flow down an inclined plane of angle θ, with free-surface elevation z=h(x,t). The pressure is hydrostatic, and the local shear stress varies linearly across the depth, so that

28 $$\begin{aligned} \begin{aligned} p(x,z,t)&=p_a+\rho _m g\cos \theta \left( h-z\right) \\ \tau _{xz}(x,z,t)&=\rho _m g\left( h-z\right) \left( \sin \theta -\cos \theta \frac{\partial h}{\partial x} \right) \end{aligned} \end{aligned}$$

and the basal shear stress is

29 $$\begin{aligned} \tau _b(x,t) = \tau _{xz}(x,0,t) = \rho _m g h \left( \sin \theta -\cos \theta \frac{\partial h}{\partial x} \right) \end{aligned}$$

where $\rho _m$ is the density of the granular–fluid mixture.

**Fig. 13:**
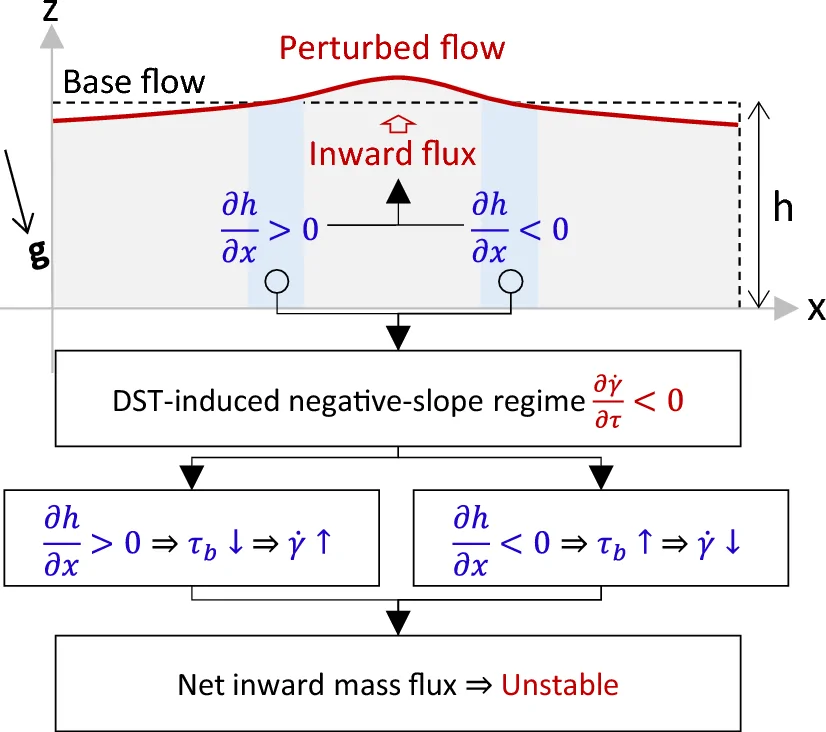
Instability mechanism

Equation (29) reveals the positive-feedback mechanism. A local increase in flow thickness reduces $\tau _b$ upstream and increases $\tau _b$ downstream through the hydrostatic-pressure-gradient term. In the DST-induced negative-slope regime, $\partial \dot{\gamma }/\partial \tau <0$, a decrease in shear stress increases the local shear rate, whereas an increase in shear stress decreases it. This generates an inward mass flux towards the perturbation and amplifies the initial free-surface disturbance, as shown in Fig. 13.

Using the local closure $\dot{\gamma }=\partial u/\partial z=\Gamma (\tau _{xz};\Phi )$, the depth-integrated discharge can be reduced to $q(x,t)=h^2\,G(\tau _b;\Phi )$, where the depth-averaged constitutive response is defined as

30 $$\begin{aligned} G(\tau _b;\Phi ) := \frac{1}{\tau _b^2} \int _{0}^{\tau _b} \tau \,\Gamma (\tau ;\Phi )\,d\tau \end{aligned}$$

We now consider a steady uniform base flow of thickness $h_0$, basal shear stress $\tau _{b0}$, discharge $q_0$, and depth-averaged velocity $u_0$, satisfying

31 $$\begin{aligned} \begin{aligned} \tau _{b0}=\rho _m g h_0\sin \theta \\ q_0=h_0^2 G(\tau _{b0};\Phi ) \\ u_0=h_0 G(\tau _{b0};\Phi ) \end{aligned} \end{aligned}$$

The free-surface evolution follows the depth-integrated mass conservation equation

32 $$\begin{aligned} \frac{\partial h}{\partial t} + \frac{\partial (hu)}{\partial x} = 0 \end{aligned}$$

Introducing infinitesimal perturbations about the uniform base state, $h=h_0+\hat{h}$, $u=u_0+\hat{u}$, and $\tau _b=\tau _{b0}+\hat{\tau }$, and linearizing Eq. (32) together with the constitutive relation $u/h=G(\tau _b;\Phi )$, one obtains

33 $$\begin{aligned} \frac{\partial \hat{h}}{\partial t} + c_0 \frac{\partial \hat{h}}{\partial x} = D_0 \frac{\partial ^2 \hat{h}}{\partial x^2} \end{aligned}$$

with

34 $$\begin{aligned} \begin{aligned} c_0&= 2u_0+\rho _m g h_0^2\sin \theta \,G_0' \\ D_0&= \rho _m g h_0^3\cos \theta \,G_0' \\ G_0' :&= \left. \frac{\partial G(\tau _b;\Phi )}{\partial \tau _b} \right| _{\tau _b=\tau _{b0}} \end{aligned} \end{aligned}$$

Equation (33) shows that perturbations are diffusive when $D_0>0$ and anti-diffusive when $D_0<0$. Since $\rho _m>0$, $h_0>0$, and $\cos \theta >0$ for $0<\theta <\pi /2$, the stability condition is governed entirely by the sign of $G_0'$, namely

35 $$\begin{aligned} G_0' < 0 \quad \Longleftrightarrow \quad \text {long-wave instability} \end{aligned}$$

Introducing the dimensionless variables $\tilde{x}=x/h_0$, $\tilde{t}=u_0 t/h_0$, $\tilde{\tau }_b=\tau _b/\tau _{b0}$, and $\tilde{\bar{\dot{\gamma }}}=\bar{\dot{\gamma }}/\bar{\dot{\gamma }}_0$, the stability problem can be expressed through the single constitutive slope parameter

36 $$\begin{aligned} A_0 := \left. \frac{d\tilde{\bar{\dot{\gamma }}}}{d\tilde{\tau }_b} \right| _{\tilde{\tau }_b=1} = \frac{\tau _{b0}}{G(\tau _{b0};\Phi )}\,G_0' \end{aligned}$$

The resulting dimensionless long-wave equation becomes

37 $$\begin{aligned} \frac{\partial h_1}{\partial \tilde{t}} + \left( 2+A_0\right) \frac{\partial h_1}{\partial \tilde{x}} = \frac{A_0}{\tan \theta } \frac{\partial ^2 h_1}{\partial \tilde{x}^2} \end{aligned}$$

so that a normal mode $h_1=\hat{h}\exp (s\tilde{t}+ik\tilde{x})$ yields the dispersion relation

38 $$\begin{aligned} s = -i\left( 2+A_0\right) k - \frac{A_0}{\tan \theta }k^2, \qquad \Re (s) = -\frac{A_0}{\tan \theta }k^2 \end{aligned}$$

Hence, the long-wave perturbation decays for $A_0>0$, is neutrally stable for $A_0=0$, and grows for $A_0<0$.

The present depth-averaged analysis is conditional on the existence of an admissible macro-viscous flowing base state. Within this simplified framework, the onset of instability is governed by the sign of the depth-averaged constitutive slope. The most relevant variables for stability classification are therefore Φ and $\tau _{b0}$, whereas the inclination angle θ enters mainly through the geometric relation $\tau _{b0}=\rho _m g h_0\sin \theta$ and through the factor $1/\tan \theta$ in the growth rate.

**Fig. 14:**
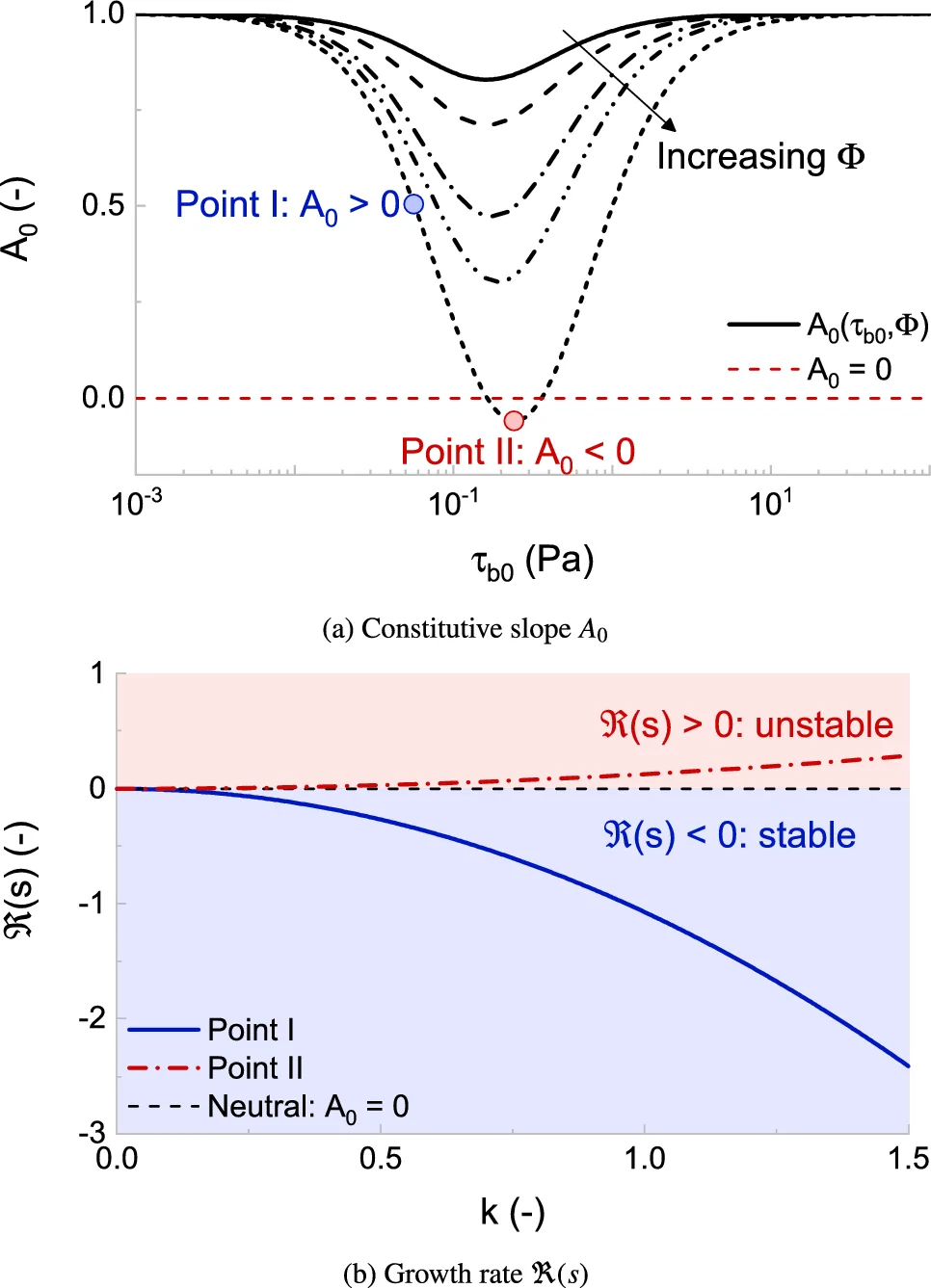
Stability criterion and dispersion behaviour in the macro-viscous limiting case

**Fig. 15:**
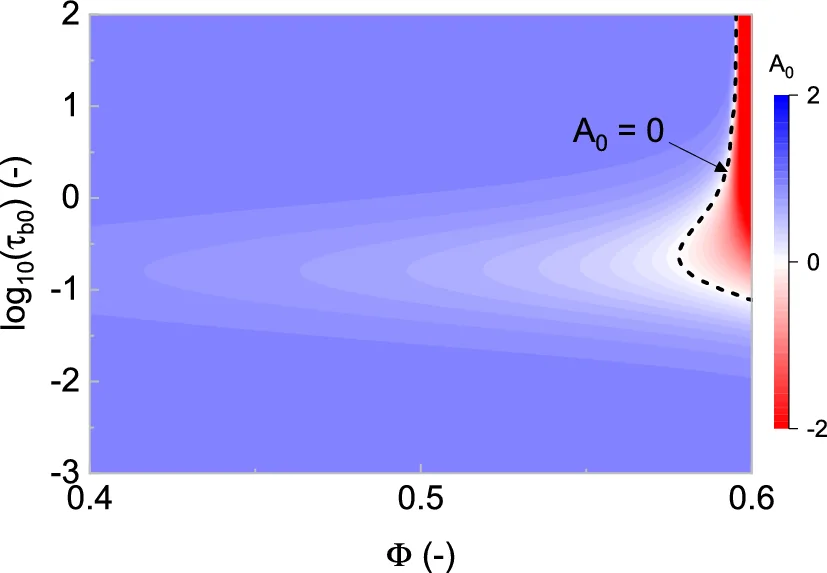
Stability map in the $(\Phi ,\tau _{b0})$ plane based on the constitutive slope parameter $A_0$

Figures 14 and 15 show how this constitutive criterion translates into free-surface stability behaviour. In dimensionless form, the stability condition is controlled by the sign of the parameter $A_0$. A positive value of $A_0$ corresponds to a diffusive response, for which free-surface perturbations are smoothed out, whereas a negative value of $A_0$ implies an anti-diffusive response and hence long-wave instability. A negative value of $A_0$ is the depth-averaged counterpart of the local negative-slope rheology. From Eq. (36), $A_0=d\tilde{\bar{\dot{\gamma }}}/d\tilde{\tau }_b|_{\tilde{\tau }_b=1}<0$ means that an increase in basal shear stress reduces the depth-averaged shear rate. Combined with the basal-stress variation induced by a free-surface perturbation in Eq. (29), this negative-slope response produces the inward-flux feedback illustrated schematically in Fig. 13, thereby amplifying free-surface perturbations. In this macro-viscous limiting case, the instability is therefore constitutive in origin and does not require any inertial mechanism.

Figure 14a shows the variation of $A_0$ with basal shear stress for different solid fractions. At relatively low Φ, $A_0$ remains positive over the whole stress range considered, indicating that the corresponding shallow flows are linearly stable. As Φ increases, however, a finite interval with $A_0<0$ emerges, showing that dense states may enter a regime in which the depth-averaged constitutive response becomes negatively sloped. The neutral points $A_0=0$ therefore mark the onset of constitutive instability. The consequence for perturbation growth is shown in Fig. 14b through the dispersion relation. For a base state with $A_0>0$ (Point I), the real part of the growth rate remains negative and disturbances decay. For a base state with $A_0<0$ (Point II), ℜ(s) becomes positive within the long-wave range considered, indicating anti-diffusive amplification. The neutral case $A_0=0$ separates these two regimes. This confirms that the sign of $A_0$ alone is sufficient to classify the linear stability of the macro-viscous shallow flow.

Figure 15 summarises the stability landscape in the $(\Phi ,\tau _{b0})$ plane. The neutral contour $A_0=0$ separates stable and unstable regions and shows that instability is restricted to relatively dense states within an intermediate range of basal stress. Outside this band, the constitutive slope remains positive and the corresponding free-surface response is diffusive. The map thus demonstrates that the non-monotonicity of the constitutive relation survives depth averaging and remains active at the shallow-flow scale.

## Conclusions

This study develops a simple constitutive framework for thinning–thickening transitions in dense granular–fluid mixtures by decomposing the effective granular stress into viscoplastic structural and suspension-stress contributions. The main conclusions are:

1. The thinning–thickening transition is governed by the changing relative contributions of the two effective-stress components. The viscoplastic structural stress controls residual/yield resistance and apparent shear thinning at low shear rates, whereas the suspension stress becomes dominant as shearing develops. Stress-activated frictional contacts and the associated evolution of the jamming state give rise to CST and DST.
2. The benchmark cases show that both DST and non-DST responses can be reproduced within the same constitutive formulation by varying the corresponding material parameters, demonstrating the unified character of the framework across distinct granular–fluid rheological responses.
3. The form of the thinning–thickening response reflects the interplay among structural resistance, frictional-contact activation, and particle inertia. Finite residual/yield resistance can delay, mask, or suppress a distinct DST branch, whereas increasing particle inertia shifts the high-stress response from macro-viscous towards grain-inertial behaviour.
4. The depth-averaged analysis shows that DST-induced non-monotonicity can persist after depth averaging. In the macro-viscous limit, a negative depth-averaged constitutive slope produces anti-diffusive long-wave growth, indicating a possible constitutive origin of free-surface instability without inertia.

As a constitutive framework rather than a complete engineering model, its extension to geotechnical and geo-environmental boundary-value problems will require coupling with evolving porosity, pore-fluid flow, and spatial heterogeneity, together with further calibration and validation against well-controlled experiments.

## Data Availability

The data that support the findings of this study are available from the corresponding author upon reasonable request.

## Appendix A. Hypoplastic model with critical state

This appendix recapitulates the hypoplastic model with critical state that provides the structural backbone of the proposed formulation [55–57, 59]. The stress tensor $\boldsymbol{\upsigma }$ used here refers to the original hypoplastic stress variable; in the main text, its critical-state reduction is supplemented by a Herschel–Bulkley rheology to define the viscoplastic structural stress.

### Appendix A.1. Critical state

At the critical state, one has

A.1 $$\begin{aligned} \mathring{\boldsymbol{\upsigma }}=\textbf{0}, \qquad \textrm{tr}\textbf{D}=0, \qquad f_e=1 \end{aligned}$$

Defining the normalised strain-rate tensor as

A.2 $$\begin{aligned} \textbf{n}=\frac{\textbf{D}}{\Vert \textbf{D}\Vert } \end{aligned}$$

, it follows from $\textrm{tr}\textbf{D}=0$ that $\textrm{tr}\textbf{n}=0$, $\textbf{n}:\textbf{n}=1$.

Substituting Eq. (A.2) into the hypoplastic equation gives

A.3 $$\begin{aligned} \mathbb {L}(\boldsymbol{\upsigma }):\textbf{n} + \textbf{N}(\boldsymbol{\upsigma }) = \textbf{0} \end{aligned}$$

Using Eqs. (6) and (7), and noting that

A.4 $$\begin{aligned} (\boldsymbol{\upsigma }\otimes \textbf{I}):\textbf{n} = \boldsymbol{\upsigma }\,\textrm{tr}\textbf{n} = \textbf{0} \end{aligned}$$

Eq. (A.3) reduces to

A.5 $$\begin{aligned} (\textrm{tr}\boldsymbol{\upsigma })\,\textbf{n} + \frac{a^2}{\textrm{tr}\boldsymbol{\upsigma }} (\boldsymbol{\upsigma }:\textbf{n})\,\boldsymbol{\upsigma } + a(\boldsymbol{\upsigma }+\boldsymbol{\upsigma }_{dev}) = \textbf{0} \end{aligned}$$

Decomposing the stress tensor into its spherical and deviatoric parts,

A.6 $$\begin{aligned} \boldsymbol{\upsigma } = -\upsigma _{vol}\textbf{I} + \boldsymbol{\upsigma }_{dev}, \qquad \textrm{tr}\boldsymbol{\upsigma } = -3\upsigma _{vol} \end{aligned}$$

and substituting Eq. (A.6) into Eq. (A.5), one obtains

A.7 $$\begin{aligned} -3\upsigma _{vol}\textbf{n} - \frac{a^2}{3\upsigma _{vol}} (\boldsymbol{\upsigma }:\textbf{n})\,\boldsymbol{\upsigma } + a(-\upsigma _{vol}\textbf{I}+2\boldsymbol{\upsigma }_{dev}) = \textbf{0} \end{aligned}$$

Taking the trace of Eq. (A.7) and using

A.8 $$\begin{aligned} \textrm{tr}\textbf{n}=0, \qquad \textrm{tr}\boldsymbol{\upsigma }=-3\upsigma _{vol}, \qquad \textrm{tr}\boldsymbol{\upsigma }_{dev}=0 \end{aligned}$$

yield

A.9 $$\begin{aligned} a^2(\boldsymbol{\upsigma }:\textbf{n})-3a\upsigma _{vol}=0 , \end{aligned}$$

that is,

A.10 $$\begin{aligned} \boldsymbol{\upsigma }:\textbf{n} = \frac{3\upsigma _{vol}}{a} \end{aligned}$$

Substituting Eq. (A.10) back into Eq. (A.7) and retaining the deviatoric part give

A.11 $$\begin{aligned} -3\upsigma _{vol}\textbf{n} - a\boldsymbol{\upsigma }_{dev} + 2a\boldsymbol{\upsigma }_{dev} = \textbf{0} \end{aligned}$$

from which it follows that

A.12 $$\begin{aligned} \boldsymbol{\upsigma }_{dev} = \frac{3\upsigma _{vol}}{a}\,\textbf{n} \end{aligned}$$

Equation (A.12) shows that the deviatoric stress tensor is collinear with $\textbf{n}$ at the critical state. Since $\textrm{tr}\textbf{D}=0$, one has $\textbf{D}_{dev}=\textbf{D}$, and therefore, $\textbf{n}$ may be equivalently defined from either $\textbf{D}$ or $\textbf{D}_{dev}$.

Defining the equivalent shear stress as

A.13 $$\begin{aligned} \tau _{\upsigma } = \sqrt{ \frac{1}{2} \boldsymbol{\upsigma }_{dev}:\boldsymbol{\upsigma }_{dev} }= \sqrt{ \frac{1}{2} \left( \frac{3\upsigma _{vol}}{a} \right) ^2 (\textbf{n}:\textbf{n}) } = \frac{3\upsigma _{vol}}{\sqrt{2}a} \end{aligned}$$

The relationship between the deviatoric stress tensor and the deviatoric strain-rate tensor is given as

A.14 $$\begin{aligned} \frac{\boldsymbol{\upsigma }_{dev}}{\tau _{\upsigma }} = \frac{\dot{\textbf{e}}}{\dot{\gamma } /2} \end{aligned}$$

Hence, the stress state at the critical state can be expressed as

A.15 $$\begin{aligned} \boldsymbol{\upsigma } = \frac{3\sqrt{2}\upsigma _{vol}}{a \dot{\gamma }} \dot{\textbf{e}} - \upsigma _{vol}\textbf{I} \end{aligned}$$

### Appendix A.2. Failure state

At the failure state, defined by $\mathring{\boldsymbol{\upsigma }}=\textbf{0}$, the expression $\textbf{n}:\textbf{n}=1$ can be rewritten as $\textbf{H}:\textbf{H}=1$, with $\textbf{H}$ given as

A.16 $$\begin{aligned} \begin{aligned} \textbf{H}(\boldsymbol{\upsigma })=&-\frac{f_e a}{\textrm{tr}\boldsymbol{\upsigma }} \left( \boldsymbol{\upsigma }+\boldsymbol{\upsigma }_{dev} \right) \\&+\frac{f_e a \boldsymbol{\upsigma }}{\textrm{tr}\boldsymbol{\upsigma }} \frac{ \left( f_v+\dfrac{a^2}{\left( \textrm{tr}\boldsymbol{\upsigma }\right) ^2} \left( \boldsymbol{\upsigma }:\boldsymbol{\upsigma } +\boldsymbol{\upsigma }:\boldsymbol{\upsigma }_{dev}\right) \right) }{ 1+f_v+\dfrac{a^2}{\left( \textrm{tr}\boldsymbol{\upsigma }\right) ^2} (\boldsymbol{\upsigma }:\boldsymbol{\upsigma }) } \end{aligned} \end{aligned}$$

We define the dimensionless invariant ratio

A.17 $$\begin{aligned} x = \frac{ \boldsymbol{\upsigma }_{dev}:\boldsymbol{\upsigma }_{dev} }{ \left( \textrm{tr}\boldsymbol{\upsigma }\right) ^2 } \end{aligned}$$

Using

A.18 $$\begin{aligned} \boldsymbol{\upsigma }:\boldsymbol{\upsigma } = \boldsymbol{\upsigma }_{dev}:\boldsymbol{\upsigma }_{dev} + \frac{\left( \textrm{tr}\boldsymbol{\upsigma }\right) ^2}{3}, \qquad \boldsymbol{\upsigma }:\boldsymbol{\upsigma }_{dev} = \boldsymbol{\upsigma }_{dev}:\boldsymbol{\upsigma }_{dev} , \end{aligned}$$

the tensor $\textbf{H}$ can be reduced to

A.19 $$\begin{aligned} \textbf{H}= \frac{f_e a}{\textrm{tr}\boldsymbol{\upsigma }} \left[ (R-2)\boldsymbol{\upsigma }_{dev} + \frac{R-1}{3}\, \left( \textrm{tr}\boldsymbol{\upsigma }\right) \textbf{I}\right] \end{aligned}$$

where

A.20 $$\begin{aligned} R= \frac{ f_v+a^2\left( 2x+\dfrac{1}{3}\right) }{ 1+f_v+a^2\left( x+\dfrac{1}{3}\right) } \end{aligned}$$

Substituting Eq. (A.19) into $\textbf{H}:\textbf{H}=1$ gives a scalar equation in x,

A.21 $$\begin{aligned} B_0x^2+B_1x+B_2=0 \end{aligned}$$

with

A.22 $$\begin{aligned} \begin{aligned} B_0&=a^4\left( \frac{f_e^2a^2}{3}-1\right) \\ B_1&=a^2\left[ f_e^2\left( (B_3+1)^2-\frac{2a^2}{3}\right) -2B_3\right] \\ B_2&=\frac{f_e^2a^2}{3}-B_3^2 \\ B_3&=1+f_v+\frac{a^2}{3} \end{aligned} \end{aligned}$$

Let $x_* \ge 0$ denote the admissible real non-negative root of Eq. (A.21). It is given by

A.23 $$\begin{aligned} x_*= \left\{ \begin{array}{cc} \dfrac{-B_1 + \sqrt{B_1^2 - 4 B_0 B_2}}{2 B_0} &  \text {if } B_0 \ne 0 \\ -\dfrac{B_2}{B_1} &  \text {if } B_0 = 0 \end{array} \right. \end{aligned}$$

Then,

A.24 $$\begin{aligned} \boldsymbol{\upsigma }_{dev}:\boldsymbol{\upsigma }_{dev} = x_* \left( \textrm{tr}\boldsymbol{\upsigma }\right) ^2 = 9x_*\upsigma _{vol}^2 \end{aligned}$$

which leads to

A.25 $$\begin{aligned} \tau _{\upsigma } = 3\sqrt{\frac{x_*}{2}}\,\upsigma _{vol} \end{aligned}$$

Therefore, the original criterion is exactly equivalent to the Drucker–Prager-type form

A.26 $$\begin{aligned} F(\boldsymbol{\upsigma }) = \tau _{\upsigma } - \alpha _{DP}\,\upsigma _{vol} =0 \end{aligned}$$

with

A.27 $$\begin{aligned} \alpha _{DP} = 3\sqrt{\frac{x_*}{2}} \end{aligned}$$

### Appendix A.3. Numerical implementation

Given a trial stress tensor $\boldsymbol{\upsigma }^{tr}$, we first compute

A.28 $$\begin{aligned} \upsigma _{vol}^{tr} = -\frac{1}{3}\,\textrm{tr}\boldsymbol{\upsigma }^{tr}, \qquad \boldsymbol{\upsigma }_{dev}^{tr} = \boldsymbol{\upsigma }^{tr} + \upsigma _{vol}^{tr}\textbf{I}\end{aligned}$$

where the volumetric stress is kept unchanged during the radial return, i.e. $\upsigma _{vol}^{n+1}=\upsigma _{vol}^{tr}$.

The corresponding equivalent deviatoric stress is

A.29 $$\begin{aligned} \tau _{\upsigma }^{tr} = \sqrt{ \frac{1}{2} \boldsymbol{\upsigma }_{dev}^{tr}:\boldsymbol{\upsigma }_{dev}^{tr} }. \end{aligned}$$

The yield function evaluated at the trial state reads

A.30 $$\begin{aligned} F^{tr} = \tau _{\upsigma }^{tr} - \alpha _{DP}\,\upsigma _{vol}^{tr} \end{aligned}$$

Using the above equation, the stress state can be updated by

A.31 $$\begin{aligned} \boldsymbol{\upsigma }^{n+1}= \left\{ \begin{array}{ll} \boldsymbol{\upsigma }^{tr} &  \text {if } F^{tr} \le 0\\ - \upsigma _{vol}^{n+1}\textbf{I}+ \frac{ \alpha _{DP}\,\upsigma _{vol}^{tr} }{ \tau _{\upsigma }^{tr} } \boldsymbol{\upsigma }_{dev}^{tr} &  \text {if } F^{tr} > 0 \end{array} \right. \end{aligned}$$

which satisfies

A.32 $$\begin{aligned} F(\boldsymbol{\upsigma }^{n+1})=0 \end{aligned}$$

## Appendix B. Depth averaging of the local closure

### Appendix B.1. Local shear stress and shear rate

We consider a two-dimensional shallow free-surface flow down an inclined plane. Under the long-wave and hydrostatic approximations, the local shear stress varies linearly across the depth,

B.1 $$\begin{aligned} \tau _{xz}(x,z,t) = \rho _m g\left( h-z\right) \left( \sin \theta -\cos \theta \frac{\partial h}{\partial x} \right) \end{aligned}$$

so that the basal shear stress is

B.2 $$\begin{aligned} \tau _b(x,t) = \tau _{xz}(x,0,t) = \rho _m g h \left( \sin \theta -\cos \theta \frac{\partial h}{\partial x} \right) \end{aligned}$$

Since the flow is unidirectional in the present lubrication setting,

B.3 $$\begin{aligned} \dot{\gamma }(x,z,t)=\frac{\partial u}{\partial z} = \Gamma \!\left( \tau _{xz}(x,z,t);\Phi \right) \end{aligned}$$

For fixed (x,t), define

B.4 $$\begin{aligned} K(x,t) = \rho _m g \left( \sin \theta -\cos \theta \frac{\partial h}{\partial x} \right) = \frac{\tau _b}{h} \end{aligned}$$

so that

B.5 $$\begin{aligned} \tau _{xz}(x,z,t)=K(h-z) \end{aligned}$$

Using the no-slip condition u(x,0,t)=0, the velocity profile is

B.6 $$\begin{aligned} u(x,z,t) = \int _0^z \Gamma \!\left( K(h-s);\Phi \right) \,ds \end{aligned}$$

Introducing τ=K(h-s), this may be rewritten as

B.7 $$\begin{aligned} u(x,z,t) = \frac{1}{K} \int _{\tau _{xz}(x,z,t)}^{\tau _b(x,t)} \Gamma (\tau ;\Phi )\,d\tau \end{aligned}$$

The depth-integrated discharge is

B.8 $$\begin{aligned} q(x,t) = \int _0^h u(x,z,t)\,dz = \int _0^h (h-s)\, \Gamma \!\left( K(h-s);\Phi \right) \,ds \end{aligned}$$

Using again τ=K(h-s), one obtains

B.9 $$\begin{aligned} q(x,t) = \frac{h^2}{\tau _b^2} \int _0^{\tau _b} \tau \,\Gamma (\tau ;\Phi )\,d\tau \end{aligned}$$

Defining the depth-averaged shear rate as

B.10 $$\begin{aligned} \bar{\dot{\gamma }} = \frac{q}{h^2} , \end{aligned}$$

the depth-averaged constitutive relation follows as

B.11 $$\begin{aligned} \bar{\dot{\gamma }} = G(\tau _b;\Phi ) := \frac{1}{\tau _b^2} \int _0^{\tau _b} \tau \,\Gamma (\tau ;\Phi )\,d\tau \end{aligned}$$

It is convenient to introduce

B.12 $$\begin{aligned} \mathcal {J}(\tau _b;\Phi ) := \int _0^{\tau _b} \tau \,\Gamma (\tau ;\Phi )\,d\tau , \qquad G(\tau _b;\Phi )=\frac{\mathcal {J}(\tau _b;\Phi )}{\tau _b^2} \end{aligned}$$

from which differentiation gives

B.13 $$\begin{aligned} G'(\tau _b;\Phi ) = \frac{\Gamma (\tau _b;\Phi )}{\tau _b} - \frac{2}{\tau _b^3}\mathcal {J}(\tau _b;\Phi ) \end{aligned}$$

### Appendix B.2. Linearisation and dispersion relation

We consider a steady uniform base flow $(h_0,\tau _{b0},q_0,u_0)$ satisfying

B.14 $$\begin{aligned} \begin{aligned} \tau _{b0}&=\rho _m g h_0\sin \theta \\ q_0&=h_0^2 G(\tau _{b0};\Phi ) \\ u_0&=h_0 G(\tau _{b0};\Phi ) \end{aligned} \end{aligned}$$

The thickness evolution follows

B.15 $$\begin{aligned} \frac{\partial h}{\partial t} + \frac{\partial (hu)}{\partial x} = 0 \end{aligned}$$

Introducing infinitesimal perturbations

B.16 $$\begin{aligned} h=h_0+\hat{h}, \qquad u=u_0+\hat{u}, \qquad \tau _b=\tau _{b0}+\hat{\tau } \end{aligned}$$

and linearizing Eq. (B.15), one obtains

B.17 $$\begin{aligned} \frac{\partial \hat{h}}{\partial t} + h_0\frac{\partial \hat{u}}{\partial x} + u_0\frac{\partial \hat{h}}{\partial x} = 0 \end{aligned}$$

Linearisation of Eq. (B.2) about the base state gives

B.18 $$\begin{aligned} \hat{\tau } = \rho _m g\sin \theta \,\hat{h} - \rho _m g h_0\cos \theta \, \frac{\partial \hat{h}}{\partial x} \end{aligned}$$

Since the depth-averaged constitutive relation is $u/h=G(\tau _b;\Phi )$, its linearisation yields

B.19 $$\begin{aligned} \hat{u} - \frac{u_0}{h_0}\hat{h} = h_0 G_0'\,\hat{\tau }, \qquad G_0' := \left. \frac{\partial G(\tau _b;\Phi )}{\partial \tau _b} \right| _{\tau _b=\tau _{b0}} \end{aligned}$$

Differentiating Eq. (B.19) with respect to x, and using Eq. (B.18), gives

B.20 $$\begin{aligned} \frac{\partial \hat{u}}{\partial x} = \left( \frac{u_0}{h_0} + \rho _m g h_0 \sin \theta \,G_0' \right) \frac{\partial \hat{h}}{\partial x} - \rho _m g h_0^2 \cos \theta \,G_0' \frac{\partial ^2 \hat{h}}{\partial x^2} \end{aligned}$$

Substituting Eq. (B.20) into Eq. (B.17), one obtains

B.21 $$\begin{aligned} \frac{\partial \hat{h}}{\partial t} + c_0\frac{\partial \hat{h}}{\partial x} = D_0\frac{\partial ^2 \hat{h}}{\partial x^2} \end{aligned}$$

with

B.22 $$\begin{aligned} c_0 = 2u_0+\rho _m g h_0^2\sin \theta \,G_0', \qquad D_0 = \rho _m g h_0^3\cos \theta \,G_0' \end{aligned}$$

Introducing the dimensionless variables $\tilde{x}=x/h_0$, $\tilde{t}=u_0 t/h_0$, $\tilde{\tau }_b=\tau _b/\tau _{b0}$, $\tilde{\bar{\dot{\gamma }}}=\bar{\dot{\gamma }}/\bar{\dot{\gamma }}_0$, together with $h=h_0(1+h_1)$, $u=u_0(1+u_1)$, and $\tau _b=\tau _{b0}(1+\tau _1)$, the constitutive slope parameter becomes

B.23 $$\begin{aligned} A_0 := \left. \frac{d\tilde{\bar{\dot{\gamma }}}}{d\tilde{\tau }_b} \right| _{\tilde{\tau }_b=1} = \frac{\tau _{b0}}{G(\tau _{b0};\Phi )}\,G_0' \end{aligned}$$

The corresponding dimensionless long-wave equation is

B.24 $$\begin{aligned} \frac{\partial h_1}{\partial \tilde{t}} + \left( 2+A_0\right) \frac{\partial h_1}{\partial \tilde{x}} = \frac{A_0}{\tan \theta } \frac{\partial ^2 h_1}{\partial \tilde{x}^2} \end{aligned}$$

Assuming a normal mode $h_1=\hat{h}\exp (s\tilde{t}+ik\tilde{x})$, one obtains

B.25 $$\begin{aligned} s = -i\left( 2+A_0\right) k - \frac{A_0}{\tan \theta }k^2, \qquad \Re (s) = -\frac{A_0}{\tan \theta }k^2 \end{aligned}$$

Hence, $A_0>0$ implies decay of long-wave perturbations, $A_0=0$ defines the neutral condition, and $A_0<0$ implies long-wave growth.
